# The role of interleukin-10 receptor alpha (*IL10Rα*) in *Mycobacterium avium* subsp. *paratuberculosis* infection of a mammary epithelial cell line

**DOI:** 10.1186/s12863-024-01234-w

**Published:** 2024-06-12

**Authors:** Aisha Fong, Christina M. Rochus, Umesh K. Shandilya, Maria M.M. Muniz, Ankita Sharma, Flavio S. Schenkel, Niel A. Karrow, Christine F. Baes

**Affiliations:** 1 Department of Animal Biosciences, Centre for Genetic Improvement of Livestock, University of Guelph, Guelph, ON N1G 2W1 Canada; 2 Present Address: The Roslin Institute, The Royal (Dick) School of Veterinary Studies, The University of Edinburgh, Easter Bush Campus, Midlothian, EH25 9RG UK; 3 Institute of Genetics, Vetsuisse Faculty, University of Bern, Bern, 3002 Switzerland

**Keywords:** Differentially expressed genes, *Interleukin-10 receptor subunit alpha*, Johne’s disease, *Mycobacterium avium* subspecies *paratuberculosis*, Bovine mammary epithelial cells, mRNA

## Abstract

**Background:**

Johne’s disease is a chronic wasting disease caused by the bacterium *Mycobacterium avium* subspecies *paratuberculosis* (MAP). Johne’s disease is highly contagious and MAP infection in dairy cattle can eventually lead to death. With no available treatment for Johne’s disease, genetic selection and improvements in management practices could help reduce its prevalence. In a previous study, the gene coding interleukin-10 receptor subunit alpha (IL10Rα) was associated with Johne’s disease in dairy cattle. Our objective was to determine how *IL10Rα* affects the pathogenesis of MAP by examining the effect of a live MAP challenge on a mammary epithelial cell line (MAC-T) that had *IL10Rα* knocked out using CRISPR/cas9. The wild type and the *IL10Rα* knockout MAC-T cell lines were exposed to live MAP bacteria for 72 h. Thereafter, mRNA was extracted from infected and uninfected cells. Differentially expressed genes were compared between the wild type and the *IL10Rα* knockout cell lines. Gene ontology was performed based on the differentially expressed genes to determine which biological pathways were involved.

**Results:**

Immune system processes pathways were targeted to determine the effect of *IL10Rα* on the response to MAP infection. There was a difference in immune response between the wild type and *IL10Rα* knockout MAC-T cell lines, and less difference in immune response between infected and not infected *IL10Rα* knockout MAC-T cells, indicating *IL10Rα* plays an important role in the progression of MAP infection. Additionally, these comparisons allowed us to identify other genes involved in inflammation-mediated chemokine and cytokine signalling, interleukin signalling and toll-like receptor pathways.

**Conclusions:**

Identifying differentially expressed genes in wild type and *ILR10α* knockout MAC-T cells infected with live MAP bacteria provided further evidence that *IL10Rα* contributes to mounting an immune response to MAP infection and allowed us to identify additional potential candidate genes involved in this process. We found there was a complex immune response during MAP infection that is controlled by many genes.

**Supplementary Information:**

The online version contains supplementary material available at 10.1186/s12863-024-01234-w.

## Background

Johne’s disease is a highly contagious chronic wasting condition caused by *Mycobacterium avium* subspecies *paratuberculosis* (MAP). Infection with MAP causes thickening of the intestines, which leads to decreased nutrient absorption, diarrhea and weight loss [1]. There is no cure, and infections eventually lead to death. A long incubation period means there is the potential for subclinical signs to be missed, such as reduced milk, protein and fat yield [2]. The disease is clinical when there is diarrhea, reduced milk production and absorption of nutrients, emaciation and death [3]. MAP is transmitted feco-orally and the most common route of transmission is contaminated colostrum and milk given to a neonatal calf; there is evidence that MAP can even survive extended pasteurization [4]. MAP can survive in the barn for up to 16 months, and both calves and cows are at risk when feces builds up from infected animals [5]. Management practices are crucial to preventing spread of MAP because they are currently the only means of control.

Dairy cattle herd-level prevalence of Johne’s disease in Ontario, Canada is an estimated 26.2–27.2%, and within-herd prevalence is an estimated 2.3–4.2% [6]. Diagnosis of Johne’s disease for dairy cattle is most commonly done with a milk ELISA test in Canada. This non-invasive test is low-cost, has a quick turn-around time, and is practical to use on-farm to get real-time results. However, the low sensitivity (30.4%) of this test leads to many false negative results [7]. These false negative results, combined with a long incubation period, create the potential for subclinical signs to be missed. Vaccines are being developed, or are in development [8–10], however, none are currently available to Canadian producers. Breeding for increased resistance to Johne’s disease could contribute to reducing its effect on individual animals and the industry.

Though low (6%), the heritability estimate for resistance to Johne’s disease indicates that selection against the disease could be an effective long-term strategy [11]. Breeding for increased resistance to Johne’s disease could contribute to the control of this disease [12], however, due to an unreliable phenotype, low sensitivity of tests and the long incubation period, it is challenging to incorporate Johne’s disease resistance into breeding programs. Despite these challenges, studies show there is genetic variation in disease susceptibility, as well as single nucleotide polymorphisms (SNPs) associated with a positive Johne’s disease test [12–25]. By knowing which genes and genetic variants are involved in resistance, they can be better represented in SNP arrays used for genotyping animals for genomic selection. By including causal variants in genomic selection, the most informative variants are used in a routine evaluation [26].

Interleukin-10 (*IL10*) is a cytokine that can regulate the antimicrobial activity of macrophages, resulting in an anti-inflammatory effect and tissue repair [27]. During an infection of murine macrophages with MAP, the IL10 receptor (IL10R) has been shown to be upregulated in response [28]. Interleukin-10 receptor alpha (IL10Rα) is one dimer making up the IL10R that binds IL10. When exposed to MAP, *IL10Rα* is upregulated, which contributes to decreasing inflammation and is thought to aid survival of MAP [29]. Four tightly linked SNPs that were discovered in *IL10Rα* were found to have an additive and dominant effect on MAP infection status [30], thus, *IL10Rα* plays a role in the progression of Johne’s disease. Precise mechanisms of this association, however, are currently not yet fully understood.

One way to learn more about the role of IL10Rα in the pathogenesis of Johne’s disease and MAP infection is by knocking out *IL10Rα* in a cell line and carrying out in vitro functional studies. An *IL10Rα* knockout cell line was created using bovine mammary epithelial (MAC-T) cells [15], and a functional study was performed by stimulating cells with MAP lysate. When comparing wild-type and knock-out MAC-T cells stimulated with MAP lysate, researchers observed increased expression of pro-inflammatory cytokines IL1α, IL1β, IL6 and TNFα in knock-out cells providing evidence that *IL10Rα* is involved in response to MAP infection [15]. This supports that genetic variation within this gene could facilitate breeding for an increased resistance to Johne’s disease. However, by infecting *IL10Rα* knockout MAC-T cells with live MAP, a response that better represents what happens in vivo will likely be observed. Therefore, the objective of this study was to examine the effect of live MAP on *IL10Rα* knockout MAC-T cells by examining differentially expressed genes between wild type MAC-T cells without exposure to MAP (WT), wild type MAC-T cells with exposure to MAP (WT-MAP), *IL10Rα* knockout MAC-T cells without exposure to MAP (KO), and *IL10Rα* knockout MAC-T cells with exposure to MAP (KO-MAP).

## Results

### Differentially expressed (DE) genes

**Fig. 1:**
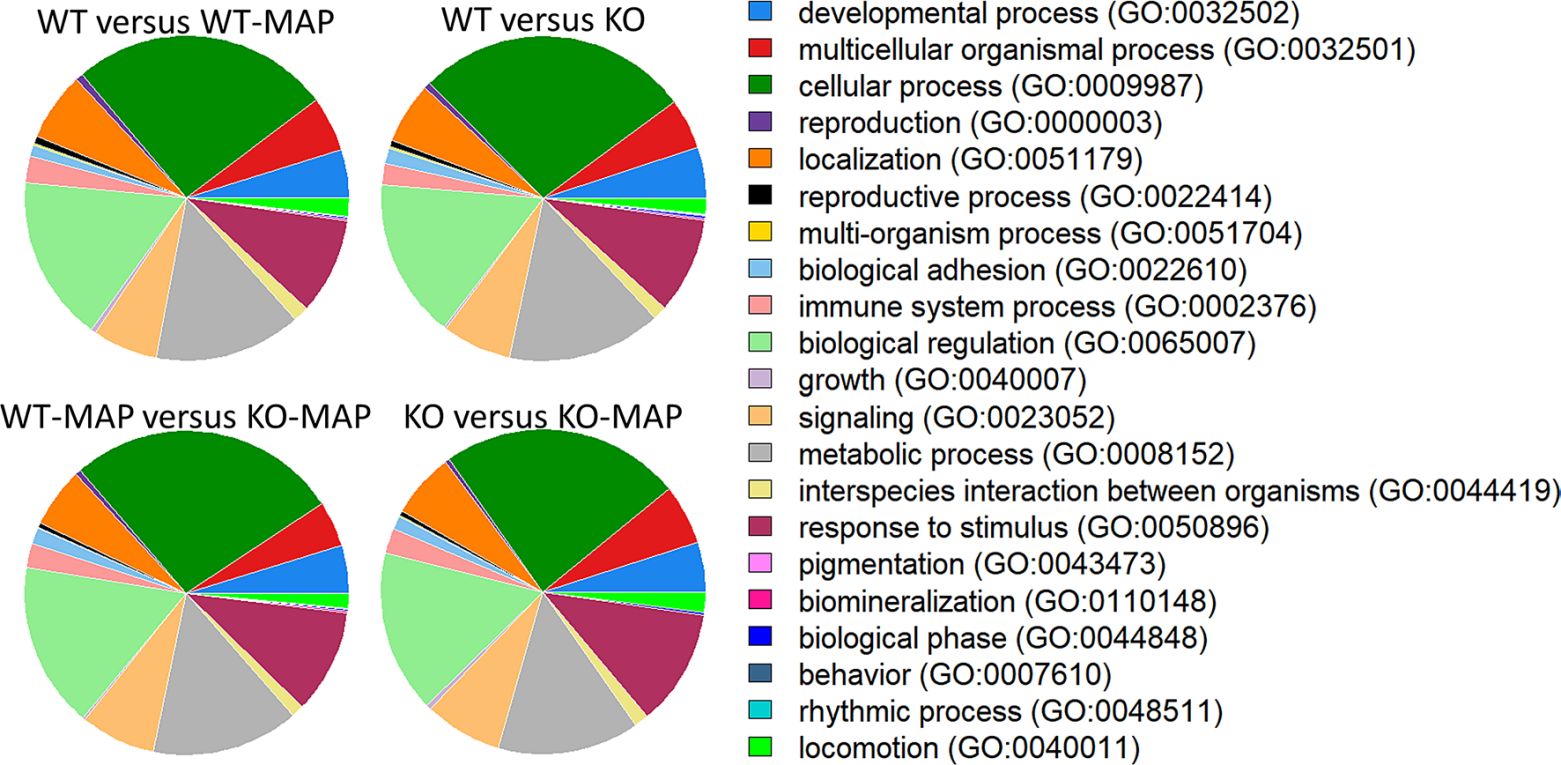
PANTHER biological processes for differentially expressed genes from the four contrasts: wildtype MAC-T cells (WT) versus the wildtype MAC-T cells infected with *Mycobacterium avium* subsp. *Paratuberculosis* (WT-MAP), WT versus the *IL10Rα*-knockout MAC-T cells (KO), WT-MAP versus the *IL10Rα*-knockout MAC-T cells infected with *Mycobacterium avium* subsp. *Paratuberculosis* (KO-MAP), and KO versus KO-MAP

In total, there were 1388 DE genes between WT and WT-MAP (Additional file 1: Table S1), 1738 DE genes between WT and KO (Additional file 1: Table S2), 1613 DE genes between WT-MAP and KO-MAP (Additional file 1: Table S3), and 561 DE genes between KO and KO-MAP (Additional file 1: Table S4). Number of shared and exclusive DE genes from the four comparisons is available in the additional materials (Additional file 2: Figure S1). We identified the 20 DE genes with the highest absolute fold change among each of the comparison groups (Additional file 3: Tables S5 to S8). None of the top 20 DE genes were identified by the PANTHER classification system as being immune system processes. The top three biological processes within all of the comparison groups were biological regulation, cellular processes, and metabolic processes (Fig. 1).

### Metabolic processes and immune response

Metabolic processes was one of the most significant gene ontology (GO) biological processes enriched by genes among all four comparisons identified in PANTHER enrichment analysis [31, 32]. The protein-protein interaction enrichment analyses were statistically significant (*p*-value < 1.0e^− 16^) for all comparison groups. A total of 46 Kyoto encyclopedia of genes and genomes (KEGG) pathways related to metabolic processes were enriched for DE genes identified through the WT versus WT-MAP contrast, 32 KEGG pathways through the WT versus KO contrast, 23 KEGG pathways through the WT-MAP versus KO-MAP contrast, and 24 KEGG pathways through the KO versus KO-MAP contrast (Additional file 4: Tables S9 to S12).

Many genes involved in immune system processes were also differentially expressed within all comparison groups even though they did not fall within the top 20 DE genes. There were six identified KEGG pathways from DE genes for the WT versus WT-MAP contrast, 11 KEGG pathways for the WT versus KO contrast, and 13 KEGG pathways for the WT-MAP versus KO-MAP contrast (Additional file 5: Tables S13 to S15). While there were 21 total DE genes relating to immune system processes for KO versus KO-MAP (Additional file 5: Table S16), no KEGG pathways were identified, possibly due to the small number of known interactions between each of the genes.

There were 213 KEGG pathways related to inflammation mediated by chemokine and cytokine signalling pathways in the four contrasts. Of these, 13 KEGG pathways were identified from the WT versus WT-MAP contrast, 98 KEGG pathways were identified from the WT versus KO contrast, and 38 were identified from the WT-MAP versus KO-MAP, and 64 KEGG pathways were identified from the KO versus KO-MAP contrast (Additional file 6: S17 to S20).

There were 132 KEGG pathways involved in interleukin signalling and toll-like receptor signalling in the four contrasts identified. Of these, 18 KEGG pathways related to interleukin signalling were identified from the WT versus WT-MAP contrast, 42 KEGG pathways from the WT versus KO contrast, 52 KEGG pathways from the WT-MAP versus KO-MAP contrast, and three KEGG pathways from the KO versus KO-MAP contrast (Additional file 7: S21 to S24). Additionally, 17 KEGG pathways related to toll-like receptor signalling were identified from the WT versus WT-MAP contrast (Additional file 8: 25). No other contrasts identified the toll-like receptor signalling KEGG pathway.

**Fig. 2:**
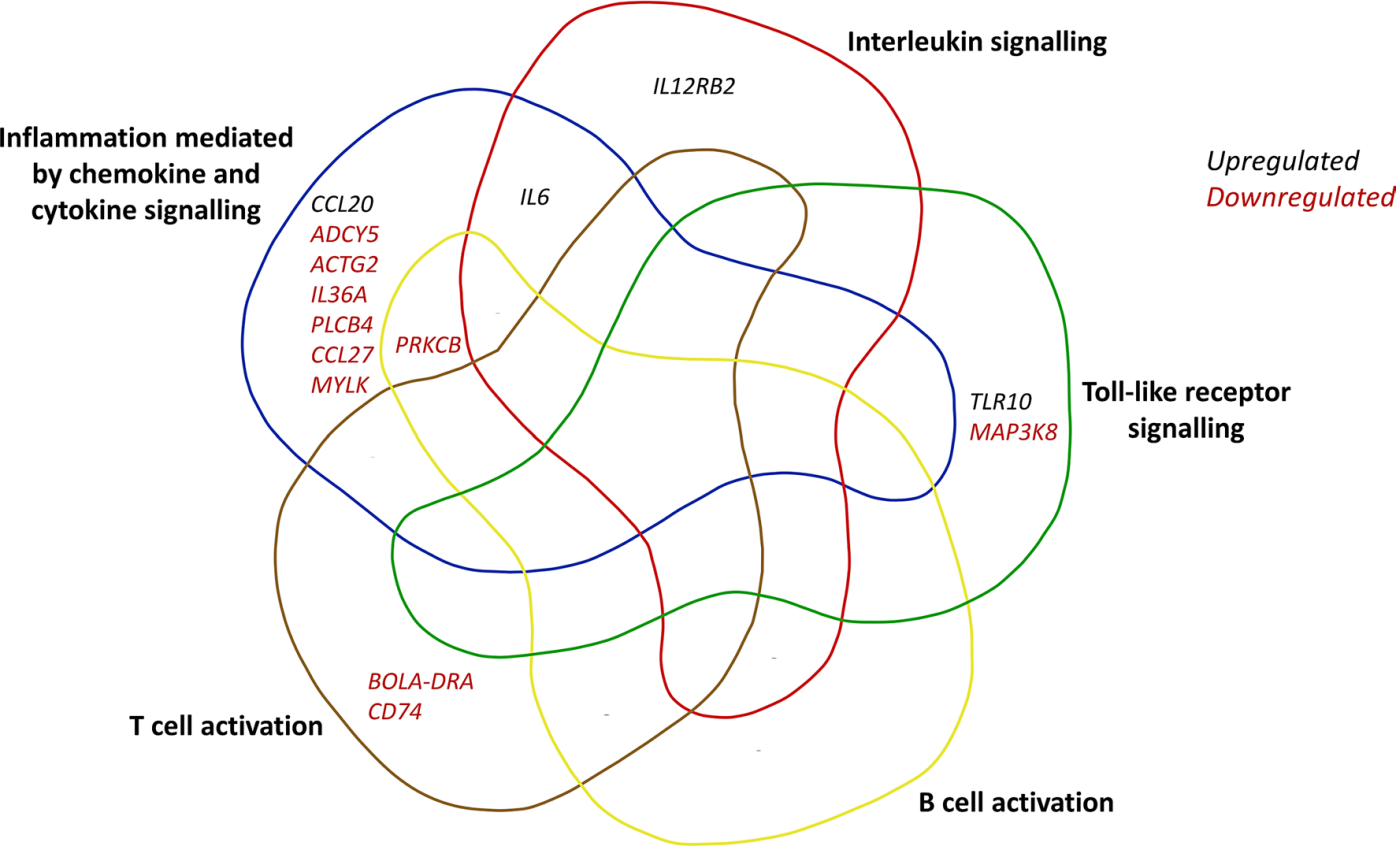
PANTHER immune system processes for differentially expressed genes from the contrast of the *IL10Rα*-knockout MAC-T cells (KO) versus the *IL10Rα*-knockout MAC-T cells infected with *Mycobacterium avium* subsp. *Paratuberculosis* (KO-MAP)

Overall, there was little difference in immune response in the KO versus KO-MAP contrast. There were 21 DE genes related to immune system processes (Additional file 5: Table S16), 9 DE genes related to inflammation (Additional file 9: Table S26), and 2 DE genes related to the interleukin signalling pathway (Additional file 9: Table S27). Protein-Protein interaction analyses was performed in STRING [33] which revealed significant enrichment for the genes related to immune system processes (Additional file 10). However, there was no enrichment for DE genes involved in toll-like receptor and interleukin signalling pathways. Figure 2 shows all DE genes from the KO versus KO-MAP contrast involved in inflammation mediated by chemokine and cytokine signalling, interleukin signalling, toll-like receptor signalling, B cell activation, and T cell activation pathways. The majority of these genes were downregulated in the KO-MAP treatment, and most are exclusive to inflammation and no other immune system function.

## Discussion

In this knockout study, we investigated the MAC-T cell response to MAP infection in the absence of *IL10Rα*. Our results highlighted the roles of metabolic and immune responses to MAP infection, specifically identifying genes involved in inflammation mediated chemokine and cytokine signalling, interleukin signalling and toll-like receptor pathways. This study has provided further evidence of the role of *IL10Rα* during MAP infection and has identified many other involved genes. The top three biological processes for all four comparisons were biological regulation, cellular processes, and metabolic processes (Fig. 1). These results were consistent with the literature because differences in the regulation of biological processes and cellular processes are typically observed and metabolic processes are affected in knockout gene studies [20, 34, 35], and infection with MAP bacteria has been shown to alter the metabolism of cells [36–38].

### Metabolic response

Several studies have shown lipid metabolism and cholesterol pathways were affected by MAP infection, which was likely in order to facilitate MAP survival [36–38], however, we did not specifically assess MAP viability. For macrophages, alteration of these pathways is believed to help establish infection and its persistence within intracellular compartments [36, 38]. In our study, enrichments were found in DE genes in non-infected and infected cell line comparisons, which included the KEGG pathways: cholesterol metabolism; retinol metabolism; fat digestion and absorption; and peroxisome proliferator-activated receptors (PPAR) signalling. In this study we have used MAC-T cells and not macrophages, so DE genes related to metabolism may not be due to MAP infection, but could be simply because a gene (*IL10Rα*) has been knocked-out. Additionally, enrichment analyses can be biased towards well studied genes and pathways, so our results may not convey the entirety of what is occurring.

When further examining metabolic processes among all comparisons, common genes were found in WT versus WT-MAP, WT versus KO, and WT-MAP versus KO-MAP contrasts. This shows that gene editing had an effect on MAC-T cell metabolism, because these genes were only absent in the comparison where both cell lines had *IL10Rα* knocked out, the KO versus KO-MAP comparison. Many pathways relating to adipose cells were found, such as fat digestion and absorption, cholesterol metabolism, and the adipocytokine signalling pathway (Additional file 4). Cells in the current study were mammary epithelial cells and because the mammary gland is composed of fatty tissue, and mobilization of fat is needed in the mammary gland to produce milk, the presence of pathways relating to adipose tissue is expected.

### Immune response

Immune system processes related to both innate and acquired immunity were also examined to gain a greater understanding of how *IL10Rα* affects the pathogenesis of MAP bacteria (Additional file 5). DE genes involved in the interleukin signalling pathway and the toll-like receptor signalling pathway were inspected as they play a crucial role in pathogen recognition, cell signalling and immune system activation (Additional files 7 and 8). As IL10Rα is a dimer making up the IL10 receptor for an anti-inflammatory cytokine IL10, the inflammation pathway regulated by chemokine and cytokine signalling was also examined as this pathway was expected to be significantly enriched (Additional file 6). Collectively examining DE genes involved in these pathways, as well as the B cell activation and T cell activation pathways, showed that there was an immune response due to MAP infection (Additional file 11).

In the comparison of WT versus WT-MAP, we identified DE genes involved in the immune system, including transcription factor Jun (*Jun*), which was downregulated in WT-MAP. The Jun pathway has been implicated in the activation of transcription of *IL10* in T helper 2 cells, which are a major source of IL10 [39], and MAC-T cells are also capable of producing IL10 [40]. Another identified DE gene was interleukin 6 (*IL6*), which was upregulated in WT-MAP. Amongst IL6’s pleiotropic properties, IL6 can act as a pro-inflammatory cytokine [41–43], and has been found in previous studies to be associated with IL10 and Johne’s disease [19], chronic human inflammatory lung disease [44], and bovine respiratory disease [45].

In the WT versus KO contrast, we identified DE genes involved in the immune system, such as interleukin 36 alpha (*IL36α*). *IL36α* was downregulated in the KO and encodes for a pro-inflammatory cytokine, and is a member of the interleukin 1 (IL1) family of cytokines that plays a major role in initiating inflammation in response to infection, or tissue injury [46–50]. In this comparison, mitogen activated protein kinases *(MAPK) 12* (*MAPK12*) and *MAPK13* were downregulated in the KO. These are stress-activated protein kinases that play an important role in the innate immune system signalling and inflammation [51]. Toll-like receptor 6 (*TLR6*) was upregulated in the KO, and can also activate the nuclear factor kappa beta (NF-κβ) signalling pathway to generate a pro-inflammatory response [52]. In contrast, the bovine major histocompatibility complex class II DR alpha (*BoLA-DRα*) was downregulated in the KO and *DRα* is required for antigen presentation to initiate adaptive immunity [27]. *IL6* was upregulated in the WT versus KO contrast, and all other contrasts (i.e. WT versus WT-MAP, WT-MAP versus KO-MAP, and KO versus KO-MAP).

In the comparison of WT-MAP versus KO-MAP, *IL10Rα* was downregulated in the KO-MAP, as expected, due to gene editing. Interleukin 20 receptor alpha (*IL20Rα*) was also downregulated, and it regulates the JAK-STAT signalling pathway [53, 54]. *IL20Rα* is expressed in the epidermis, which could explain its presence in MAC-T cells, however, the JAK-STAT signalling pathway is also important for immune cell development. Related to this signalling pathway, Janus kinase 3 (*JAK3*) was upregulated in the KO-MAP, and signal transducer and activator of transcription 3 (*STAT3*) was downregulated; JAK3 is involved in cytokine receptor-mediated intracellular signal transduction, and STAT3 acts as a transcription factor in response to cytokines and growth factors [54].

### Response to infection

Of the four comparisons, there were fewer DE genes identified that are associated with immune response between KO versus KO-MAP, providing evidence that *IL10Rα* is a major gene of interest when examining MAP infection. When looking at DE genes relating to inflammation, only *IL6* and chemokine (C-C motif) ligand 20 (*CCL20*) were upregulated in the KO-MAP, whereas the other seven inflammation-related genes were downregulated. *CCL20* has been shown to have increased expression in human inflammatory bowel disease [55], and is involved in the trafficking of various immune cell types [27]. Two DE genes relating to the interleukin signalling pathway were present, and these were interleukin 12 receptor beta 2 (*IL12Rβ2*), which was downregulated in the KO-MAP, and *IL6*, which was upregulated. The IL12R is involved in the activation of T cells and natural killer (NK) cells [56]. Collectively, these findings indicate that upon MAP infection, *IL10Rα* knockout cells do still respond to MAP infection, however, the response is far less than what it would be in unedited cells.

### Polygenic nature of immune response to MAP bacteria and application of results

Our results support that immune response to MAP bacteria is highly polygenic, which is expected, as genetic variation of disease-related traits is usually explained by a large number of genetic variants with small effects. An example of the highly polygenic nature of disease-related traits is chronic human inflammatory bowel disease (IBD), which has been associated with MAP infection [57, 58], where researchers have implicated more than 130 candidate genes [59–61]. Studies have shown that many genes are thought to contribute to MAP infection status [13], and incorporating resistance to Johne’s disease into genomic evaluations will involve taking into account variants in multiple genes and not just *IL10Rα*. Further research into causal variants is needed because this information could be useful in pharmaceutical development to treat Johne’s disease, and including causal variants in genotyping arrays may improve accuracy of genomic selection [26] for selecting animals more resistant to MAP infection.

## Conclusions

Johne’s disease is devastating for the dairy cattle industry, with serious animal welfare implications and substantial economic loss. Prior research identified *IL10Rα* to be involved in the immune response in MAC-T cells to MAP cell lysate, however, this may not necessarily reflect the response to a live pathogen challenge. Targeting genes involved in immune system processes allowed us to identify specific pathways that were related to DE genes between wild type and *IL10Rα* knockout MAC-T cells, such as inflammation mediated by the chemokine and cytokine signalling pathway, the interleukin signalling pathway, and the toll-like receptor signalling pathway. This study provided further information on *IL10Rα*’s role in MAP infection and pathogenesis, offering further support that *IL10Rα* is a good candidate gene, and presenting results that could help with development of therapeutics. The immune response to Johne’s disease and progression of MAP infection is complex, and this in vitro study gives further insight into other genes and pathways involved that require further study.

## Methods

### Knockout cell line development, infection, and extraction of mRNA

The MAC-T cell cultures (Nexia Biotechnologies, Quebec, Canada) were developed by Huynh et al. [62], while *IL10Rα* knockout and confirmation of knockout were performed using the same methods as Mallikarjunappa et al. [15]. MAP infections were performed using the method of Lamont et al. [63] using MAP (Madonna strain) gifted from Dr. Lucy Mutharia (University of Guelph). All the experiments were carried out in quadruplicate at four independent times. Bacteria was cultured using the methods described by Shandilya et al. [40], and the bacterial load was added to each infected treatment group to form a 10:1 multiplicity of infection [40, 64]. There were four treatment groups, described in Table 1, with unedited and *IL10Rα* knockout cells infected with MAP bacteria or not. As described in Shandilya et al. [40], MAC-T cells had MAP bacteria added (for 72 h), or for uninfected cells, MAP carrier solution media was added. Extraction of mRNA was performed using the same method as Shandilya et al. [65] using the RNeasy Mini Kit (Quiagen, Germany). Before outsourcing (Genewiz, Azenta Life Sciences, US) the samples for RNA-Sequencing, the purity of the RNA samples was determined using the Cytation 5 Spectrophotometer (Biotek, USA) at A260/280 nm ratio. The integrity of the RNA samples was assessed by bioanalyzer, and the RNA concentration was determined using the Qubit® 2.0 Fluorometer. Paired-end (2 × 150 bp) reads were generated using the Illumina HiSeq 2500 sequencer.

**Table 1:** Experimental design: MAC-T cells and *IL10Rα* knockout MAC-T cells were infected with *Mycobacterium avium* subspecies *paratuberculosis* (MAP) for 72 h, after which mRNA was extracted for sequencing

Cell type	Treatment	Group	Number of samples
Wild type MAC-T cells	Not infected	WT	4
	Infected with MAP	WT-MAP	4
*IL10Rα* knockout MAC-T cells	Not infected	KO	4
	Infected with MAP	KO-MAP	4

The data in this study have been deposited in NCBI’s Gene Expression Omnibus [66] and are accessible through GEO series accession number GSE247921 (https://www.ncbi.nlm.nih.gov/geo/query/acc.cgi?&acc=GSE247921).

### RNA-Sequencing analysis

To analyze the RNA-Sequencing data, the CLC Genomics Workbench software 20.0.4 (QIAGEN, Aarhus, Denmark) was used. FastQ files were imported into the software, and quality control was done following the parameters described in [67]. A Phred score measures the probability that a nucleobase was identified correctly during the sequencing process, with low scores indicating incorrect identification. A score of at least 20 is a standard quality control cut-off as that indicates a 99% accuracy [67]. In this study all Phred scores were above 35, and the accuracy was 99.97%. The data was then trimmed based on quality scores which included a maximum error probability of 0.05 and a maximum number of ambiguous nucleotides of 2.

For RNA-Sequencing analysis, the CLC Genomics Workbench 20.0.4 (QIAGEN, Aarhus, Denmark) environment, was used to align pair-end sequence reads of each sample against the bovine reference genome, ARS-UCD1.2 imported from Ensembl [68]. The parameters used for global alignments were 0.6 for length fraction and 0.6 for similarity fraction, which represents the minimum percentage of total alignment that must match the reference sequence with a minimum percentage identity between the aligned region of the read and the reference. In addition, two mismatches, three insertions and three deletions per read were allowed. To facilitate group comparisons, the gene levels were quantified in reads per kilobase per million mapped reads (RPKM) and transformed to log 2 [69].

Differentially expressed (DE) genes analyses for WT versus WT-MAP, WT versus KO, WT-MAP versus KO-MAP, and KO versus KO-MAP contrasts were performed using the CLC Genomics Workbench 20.0.4 (QIAGEN, Aarhus, Denmark). The two-group comparisons were carried out using samples obtained after 72 h of infection from the four groups, WT (*n*=3), WT-MAP (*n*=4), KO (*n*=4) and KO-MAP (*n*=4). The WT group only included three samples because one sample was removed due to failing the quality control. Empirical analysis was done using the DGE tool in CLC workbench and performed for each contrast, using the following parameter to keep data: total count ≥ 5.0. Transcript levels were quantified in reads per kilobase per million mapped reads (RPKM). DE genes between each contrast were defined by a p-value ≤0.01, FDR ≤0.05, and |fold change|≥2.

### Gene ontology

After DE genes were identified, GO enrichment analysis was performed for each analysed contrast, using PANTHER [31, 32]. Genes specifically related to metabolic processes, as well as the immune system processes of inflammation mediated by chemokine and cytokine signalling pathways, the Toll-like receptor signalling pathway, the interleukin signalling pathway, the B cell activation pathway, and the T cell activation pathway were targeted. Venn diagrams were created to determine overlap between each of the comparison groups [70]. Knowledge about specific targeted genes of interest was gained from GeneCards [27], and protein-protein interaction network analysis was performed using STRING [33].

### Electronic supplementary material

Below is the link to the electronic supplementary material.

Supplementary Material 1

Supplementary Material 2

Supplementary Material 3

Supplementary Material 4

Supplementary Material 5

Supplementary Material 6

Supplementary Material 7

Supplementary Material 8

Supplementary Material 9

Supplementary Material 10

Supplementary Material 11

## Data Availability

The data in this study are accessible through NCBI GEO series accession number GSE247921 (https://www.ncbi.nlm.nih.gov/geo/query/acc.cgi?&acc=GSE247921).
