# Supplementary material for: The role of interleukin-10 receptor alpha (IL10Rα) in Mycobacterium avium subsp. paratuberculosis infection of a mammary epithelial cell line
Source: BMC Genom Data. 2024 Jun 12;25:58. doi: 10.1186/s12863-024-01234-w (PMC11167801; doi:10.1186/s12863-024-01234-w)
Supplement: Supplementary file 2 — Supplementary Material 2 [file 12863_2024_1234_MOESM2_ESM.docx]

**Table S9:** KEGG pathways that were significantly enriched for differentially expressed genes involved in metabolic pathways identified from the contrast of wildtype MAC-T cells (WT) vs. the wildtype MAC-T cells infected with *Mycobacterium avium* subsp. *Paratuberculosis* (WT-MAP)

| **Term ID** | **Term Description** | **Observed Gene Count** | **Background Gene Count** | **Strength** | **False Discovery Rate** |
| --- | --- | --- | --- | --- | --- |
| bta01100 | Metabolic pathways | 59 | 1413 | 0.45 | 2.46E-10 |
| bta03320 | PPAR signaling pathway | 11 | 77 | 0.99 | 1.28E-05 |
| bta04152 | AMPK signaling pathway | 12 | 118 | 0.84 | 6.31E-05 |
| bta04668 | TNF signaling pathway | 11 | 108 | 0.84 | 0.00011 |
| bta05418 | Fluid shear stress and atherosclerosis | 12 | 128 | 0.81 | 0.00011 |
| bta04928 | Parathyroid hormone synthesis, secretion and action | 10 | 98 | 0.84 | 0.00029 |
| bta04950 | Maturity onset diabetes of the young | 6 | 26 | 1.2 | 0.00037 |
| bta01200 | Carbon metabolism | 10 | 108 | 0.8 | 0.00042 |
| bta04657 | IL-17 signaling pathway | 9 | 83 | 0.87 | 0.00042 |
| bta01230 | Biosynthesis of amino acids | 8 | 70 | 0.89 | 0.00076 |
| bta05200 | Pathways in cancer | 21 | 488 | 0.47 | 0.00076 |
| bta04926 | Relaxin signaling pathway | 10 | 121 | 0.75 | 0.00079 |
| bta00010 | Glycolysis / Gluconeogenesis | 7 | 58 | 0.92 | 0.0015 |
| bta04922 | Glucagon signaling pathway | 8 | 93 | 0.77 | 0.0035 |
| bta05161 | Hepatitis B | 10 | 158 | 0.64 | 0.0052 |
| bta00480 | Glutathione metabolism | 6 | 54 | 0.88 | 0.0064 |
| bta05162 | Measles | 9 | 135 | 0.66 | 0.0067 |
| bta01522 | Endocrine resistance | 7 | 85 | 0.75 | 0.0095 |
| bta05215 | Prostate cancer | 7 | 86 | 0.74 | 0.0096 |
| bta00072 | Synthesis and degradation of ketone bodies | 3 | 9 | 1.36 | 0.0135 |
| bta05204 | Chemical carcinogenesis | 6 | 66 | 0.79 | 0.0135 |
| bta00983 | Drug metabolism - other enzymes | 6 | 68 | 0.78 | 0.0139 |
| bta04910 | Insulin signaling pathway | 8 | 124 | 0.64 | 0.0139 |
| bta04975 | Fat digestion and absorption | 5 | 45 | 0.88 | 0.0143 |
| bta04920 | Adipocytokine signaling pathway | 6 | 70 | 0.77 | 0.0147 |
| bta04979 | Cholesterol metabolism | 5 | 46 | 0.87 | 0.0147 |
| bta05133 | Pertussis | 6 | 70 | 0.77 | 0.0147 |
| bta00030 | Pentose phosphate pathway | 4 | 27 | 1 | 0.016 |
| bta04917 | Prolactin signaling pathway | 6 | 74 | 0.74 | 0.0161 |
| bta05169 | Epstein-Barr virus infection | 10 | 201 | 0.53 | 0.0161 |
| bta04931 | Insulin resistance | 7 | 106 | 0.65 | 0.0187 |
| bta04151 | PI3K-Akt signaling pathway | 13 | 331 | 0.43 | 0.0223 |
| bta04380 | Osteoclast differentiation | 7 | 113 | 0.63 | 0.0249 |
| bta00980 | Metabolism of xenobiotics by cytochrome P450 | 5 | 58 | 0.77 | 0.0286 |
| bta05160 | Hepatitis C | 8 | 150 | 0.56 | 0.0286 |
| bta05165 | Human papillomavirus infection | 12 | 303 | 0.43 | 0.0286 |
| bta04010 | MAPK signaling pathway | 11 | 266 | 0.45 | 0.0293 |
| bta04915 | Estrogen signaling pathway | 7 | 119 | 0.6 | 0.0293 |
| bta05219 | Bladder cancer | 4 | 36 | 0.88 | 0.0298 |
| bta05231 | Choline metabolism in cancer | 6 | 89 | 0.66 | 0.0298 |
| bta00564 | Glycerophospholipid metabolism | 6 | 95 | 0.63 | 0.0385 |
| bta05218 | Melanoma | 5 | 66 | 0.71 | 0.0393 |
| bta00100 | Steroid biosynthesis | 3 | 19 | 1.03 | 0.0396 |
| bta01524 | Platinum drug resistance | 5 | 68 | 0.7 | 0.0423 |
| bta04115 | p53 signaling pathway | 5 | 68 | 0.7 | 0.0423 |
| bta05224 | Breast cancer | 7 | 135 | 0.55 | 0.0457 |

**Table S10:** KEGG pathways that were significantly enriched for differentially expressed genes involved in metabolic pathways identified from the contrast of wildtype MAC-T cells (WT) vs. the *IL10Rα*-knockout MAC-T cells (KO)

| **Term ID** | **Term Description** | **Observed Gene Count** | **Background Gene Count** | **Strength** | **False Discovery Rate** |
| --- | --- | --- | --- | --- | --- |
| bta05200 | Pathways in cancer | 32 | 488 | 0.55 | 7.13E-07 |
| bta03030 | DNA replication | 10 | 33 | 1.22 | 8.01E-07 |
| bta04110 | Cell cycle | 15 | 118 | 0.84 | 2.68E-06 |
| bta03460 | Fanconi anemia pathway | 9 | 48 | 1.01 | 8.49E-05 |
| bta04010 | MAPK signaling pathway | 19 | 266 | 0.59 | 9.97E-05 |
| bta04218 | Cellular senescence | 13 | 148 | 0.68 | 0.00055 |
| bta01100 | Metabolic pathways | 50 | 1413 | 0.29 | 0.00057 |
| bta04060 | Cytokine-cytokine receptor interaction | 18 | 283 | 0.54 | 0.00057 |
| bta05219 | Bladder cancer | 7 | 36 | 1.03 | 0.00057 |
| bta00590 | Arachidonic acid metabolism | 9 | 73 | 0.83 | 0.00075 |
| bta05218 | Melanoma | 8 | 66 | 0.82 | 0.0022 |
| bta03440 | Homologous recombination | 6 | 36 | 0.96 | 0.0032 |
| bta04014 | Ras signaling pathway | 14 | 218 | 0.55 | 0.0032 |
| bta04015 | Rap1 signaling pathway | 13 | 191 | 0.57 | 0.0032 |
| bta05206 | MicroRNAs in cancer | 11 | 147 | 0.61 | 0.0042 |
| bta04914 | Progesterone-mediated oocyte maturation | 8 | 83 | 0.72 | 0.0067 |
| bta01522 | Endocrine resistance | 8 | 85 | 0.71 | 0.0069 |
| bta04664 | Fc epsilon RI signaling pathway | 7 | 63 | 0.78 | 0.0069 |
| bta05224 | Breast cancer | 10 | 135 | 0.61 | 0.0069 |
| bta05226 | Gastric cancer | 10 | 133 | 0.61 | 0.0069 |
| bta04114 | Oocyte meiosis | 9 | 111 | 0.65 | 0.007 |
| bta04380 | Osteoclast differentiation | 9 | 113 | 0.64 | 0.0075 |
| bta05418 | Fluid shear stress and atherosclerosis | 9 | 128 | 0.58 | 0.0167 |
| bta04657 | IL-17 signaling pathway | 7 | 83 | 0.66 | 0.0226 |
| bta04350 | TGF-beta signaling pathway | 7 | 85 | 0.65 | 0.0246 |
| bta05202 | Transcriptional misregulation in cancer | 10 | 166 | 0.52 | 0.0246 |
| bta04062 | Chemokine signaling pathway | 10 | 170 | 0.51 | 0.0268 |
| bta04151 | PI3K-Akt signaling pathway | 15 | 331 | 0.39 | 0.0268 |
| bta04975 | Fat digestion and absorption | 5 | 45 | 0.78 | 0.0312 |
| bta04933 | AGE-RAGE signaling pathway in diabetic complications | 7 | 92 | 0.62 | 0.0314 |
| bta00565 | Ether lipid metabolism | 5 | 48 | 0.76 | 0.0379 |
| bta05161 | Hepatitis B | 9 | 158 | 0.49 | 0.047 |

**Table S11:** KEGG pathways that were significantly enriched for differentially expressed genes involved in metabolic pathways identified from the contrast of wildtype MAC-T cells infected with *Mycobacterium avium* subsp. *Paratuberculosis* (WT-MAP) vs. the *IL10Rα*-knockout MAC-T cells infected with *Mycobacterium avium* subsp. *Paratuberculosis* (KO-MAP)

| **Term ID** | **Term Description** | **Observed Gene Count** | **Background Gene Count** | **Strength** | **False Discovery Rate** |
| --- | --- | --- | --- | --- | --- |
| bta01100 | Metabolic pathways | 56 | 1413 | 0.36 | 3.30E-06 |
| bta03030 | DNA replication | 9 | 33 | 1.2 | 6.17E-06 |
| bta04110 | Cell cycle | 13 | 118 | 0.81 | 5.24E-05 |
| bta03460 | Fanconi anemia pathway | 7 | 48 | 0.93 | 0.0042 |
| bta04380 | Osteoclast differentiation | 10 | 113 | 0.71 | 0.0042 |
| bta05200 | Pathways in cancer | 22 | 488 | 0.42 | 0.0046 |
| bta00561 | Glycerolipid metabolism | 7 | 58 | 0.85 | 0.0071 |
| bta05166 | Human T-cell leukemia virus 1 infection | 13 | 213 | 0.55 | 0.0073 |
| bta00601 | Glycosphingolipid biosynthesis - lacto and neolacto series | 5 | 27 | 1.03 | 0.0089 |
| bta04060 | Cytokine-cytokine receptor interaction | 15 | 283 | 0.49 | 0.0089 |
| bta05206 | MicroRNAs in cancer | 10 | 147 | 0.6 | 0.0141 |
| bta04917 | Prolactin signaling pathway | 7 | 74 | 0.74 | 0.0165 |
| bta00240 | Pyrimidine metabolism | 6 | 55 | 0.8 | 0.0196 |
| bta04931 | Insulin resistance | 8 | 106 | 0.64 | 0.0229 |
| bta04657 | IL-17 signaling pathway | 7 | 83 | 0.69 | 0.0249 |
| bta05202 | Transcriptional misregulation in cancer | 10 | 166 | 0.55 | 0.0249 |
| bta04217 | Necroptosis | 9 | 144 | 0.56 | 0.0316 |
| bta04218 | Cellular senescence | 9 | 148 | 0.55 | 0.0358 |
| bta05321 | Inflammatory bowel disease | 6 | 67 | 0.72 | 0.0358 |
| bta00564 | Glycerophospholipid metabolism | 7 | 95 | 0.63 | 0.0389 |
| bta00230 | Purine metabolism | 8 | 127 | 0.56 | 0.0453 |
| bta05161 | Hepatitis B | 9 | 158 | 0.52 | 0.0453 |
| bta05418 | Fluid shear stress and atherosclerosis | 8 | 128 | 0.56 | 0.0453 |

**Table S12:** KEGG pathways that were significantly enriched for differentially expressed genes involved in metabolic pathways identified from the contrast of the *IL10Rα*-knockout MAC-T cells (KO) vs. the *IL10Rα*-knockout MAC-T cells infected with *Mycobacterium avium* subsp. *Paratuberculosis* (KO-MAP)

| **Term ID** | **Term Description** | **Observed Gene Count** | **Background Gene Count** | **Strength** | **False Discovery Rate** |
| --- | --- | --- | --- | --- | --- |
| bta01100 | Metabolic pathways | 23 | 1413 | 0.48 | 0.00098 |
| bta05161 | Hepatitis B | 7 | 158 | 0.91 | 0.0074 |
| bta05200 | Pathways in cancer | 11 | 488 | 0.62 | 0.0128 |
| bta04657 | IL-17 signaling pathway | 5 | 83 | 1.04 | 0.0133 |
| bta05169 | Epstein-Barr virus infection | 7 | 201 | 0.81 | 0.0133 |
| bta05160 | Hepatitis C | 6 | 150 | 0.87 | 0.0142 |
| bta05206 | MicroRNAs in cancer | 6 | 147 | 0.87 | 0.0142 |
| bta05164 | Influenza A | 6 | 160 | 0.84 | 0.0165 |
| bta01200 | Carbon metabolism | 5 | 108 | 0.93 | 0.0175 |
| bta04931 | Insulin resistance | 5 | 106 | 0.94 | 0.0175 |
| bta00030 | Pentose phosphate pathway | 3 | 27 | 1.31 | 0.0211 |
| bta04152 | AMPK signaling pathway | 5 | 118 | 0.89 | 0.0211 |
| bta05214 | Glioma | 4 | 64 | 1.06 | 0.0211 |
| bta05218 | Melanoma | 4 | 66 | 1.05 | 0.0211 |
| bta00051 | Fructose and mannose metabolism | 3 | 31 | 1.25 | 0.0213 |
| bta01230 | Biosynthesis of amino acids | 4 | 70 | 1.02 | 0.0213 |
| bta04920 | Adipocytokine signaling pathway | 4 | 70 | 1.02 | 0.0213 |
| bta05418 | Fluid shear stress and atherosclerosis | 5 | 128 | 0.86 | 0.0213 |
| bta03320 | PPAR signaling pathway | 4 | 77 | 0.98 | 0.0236 |
| bta00072 | Synthesis and degradation of ketone bodies | 2 | 9 | 1.61 | 0.0352 |
| bta05225 | Hepatocellular carcinoma | 5 | 154 | 0.77 | 0.0379 |
| bta04922 | Glucagon signaling pathway | 4 | 93 | 0.9 | 0.0397 |
| bta04928 | Parathyroid hormone synthesis, secretion and action | 4 | 98 | 0.87 | 0.0456 |
| bta00280 | Valine, leucine and isoleucine degradation | 3 | 47 | 1.07 | 0.0478 |
