# Supplementary material for: The role of interleukin-10 receptor alpha (IL10Rα) in Mycobacterium avium subsp. paratuberculosis infection of a mammary epithelial cell line
Source: BMC Genom Data. 2024 Jun 12;25:58. doi: 10.1186/s12863-024-01234-w (PMC11167801; doi:10.1186/s12863-024-01234-w)
Supplement: Supplementary file 3 — Supplementary Material 3 [file 12863_2024_1234_MOESM3_ESM.docx]

**Table S21:** KEGG pathways that were significantly enriched for differentially expressed genes involved in the interleukin signaling pathway identified from the contrast of wildtype MAC-T cells (WT) vs. the wildtype MAC-T cells infected with *Mycobacterium avium* subsp. *Paratuberculosis* (WT-MAP)

| **Term ID** | **Term Description** | **Observed Gene Count** | **Background Gene Count** | **Strength** | **False Discovery Rate** |
| --- | --- | --- | --- | --- | --- |
| bta04060 | Cytokine-cytokine receptor interaction | 2 | 283 | 1.67 | 0.0174 |
| bta04061 | Viral protein interaction with cytokine and cytokine receptor | 2 | 83 | 2.21 | 0.0174 |
| bta04620 | Toll-like receptor signaling pathway | 2 | 98 | 2.13 | 0.0174 |
| bta04657 | IL-17 signaling pathway | 2 | 83 | 2.21 | 0.0174 |
| bta04659 | Th17 cell differentiation | 2 | 107 | 2.1 | 0.0174 |
| bta04668 | TNF signaling pathway | 2 | 108 | 2.09 | 0.0174 |
| bta04932 | Non-alcoholic fatty liver disease | 2 | 145 | 1.96 | 0.0174 |
| bta05132 | Salmonella infection | 2 | 198 | 1.83 | 0.0174 |
| bta05133 | Pertussis | 2 | 70 | 2.28 | 0.0174 |
| bta05135 | Yersinia infection | 2 | 124 | 2.03 | 0.0174 |
| bta05142 | Chagas disease | 2 | 106 | 2.1 | 0.0174 |
| bta05161 | Hepatitis B | 2 | 158 | 1.93 | 0.0174 |
| bta05162 | Measles | 2 | 135 | 1.99 | 0.0174 |
| bta05163 | Human cytomegalovirus infection | 2 | 214 | 1.79 | 0.0174 |
| bta05166 | Human T-cell leukemia virus 1 infection | 2 | 213 | 1.8 | 0.0174 |
| bta05167 | Kaposi sarcoma-associated herpesvirus infection | 2 | 187 | 1.85 | 0.0174 |
| bta05323 | Rheumatoid arthritis | 2 | 91 | 2.17 | 0.0174 |
| bta05200 | Pathways in cancer | 2 | 488 | 1.44 | 0.0446 |

**Table S22:** KEGG pathways that were significantly enriched for differentially expressed genes involved in the interleukin signaling pathway identified from the contrast of wildtype MAC-T cells (WT) vs. the *IL10Rα*-knockout MAC-T cells (KO)

| **Term ID** | **Term Description** | **Observed Gene Count** | **Background Gene Count** | **Strength** | **False Discovery Rate** |
| --- | --- | --- | --- | --- | --- |
| bta04060 | Cytokine-cytokine receptor interaction | 6 | 283 | 1.67 | 3.18E-07 |
| bta04630 | JAK-STAT signaling pathway | 5 | 176 | 1.8 | 1.60E-06 |
| bta04061 | Viral protein interaction with cytokine and cytokine receptor | 4 | 83 | 2.03 | 6.26E-06 |
| bta04218 | Cellular senescence | 4 | 148 | 1.78 | 4.45E-05 |
| bta05161 | Hepatitis B | 4 | 158 | 1.75 | 4.60E-05 |
| bta05200 | Pathways in cancer | 5 | 488 | 1.36 | 7.83E-05 |
| bta05163 | Human cytomegalovirus infection | 4 | 214 | 1.62 | 0.00011 |
| bta05133 | Pertussis | 3 | 70 | 1.98 | 0.00019 |
| bta05321 | Inflammatory bowel disease | 3 | 67 | 2 | 0.00019 |
| bta04933 | AGE-RAGE signaling pathway in diabetic complications | 3 | 92 | 1.86 | 0.00038 |
| bta05323 | Rheumatoid arthritis | 3 | 91 | 1.86 | 0.00038 |
| bta04659 | Th17 cell differentiation | 3 | 107 | 1.79 | 0.0005 |
| bta05135 | Yersinia infection | 3 | 124 | 1.73 | 0.00071 |
| bta05162 | Measles | 3 | 135 | 1.69 | 0.00085 |
| bta04932 | Non-alcoholic fatty liver disease | 3 | 145 | 1.66 | 0.00098 |
| bta05164 | Influenza A | 3 | 160 | 1.62 | 0.0012 |
| bta04062 | Chemokine signaling pathway | 3 | 170 | 1.59 | 0.0013 |
| bta05202 | Transcriptional misregulation in cancer | 3 | 166 | 1.6 | 0.0013 |
| bta05167 | Kaposi sarcoma-associated herpesvirus infection | 3 | 187 | 1.55 | 0.0016 |
| bta05169 | Epstein-Barr virus infection | 3 | 201 | 1.52 | 0.0019 |
| bta05166 | Human T-cell leukemia virus 1 infection | 3 | 213 | 1.5 | 0.0022 |
| bta05219 | Bladder cancer | 2 | 36 | 2.09 | 0.0026 |
| bta05144 | Malaria | 2 | 48 | 1.97 | 0.0043 |
| bta05332 | Graft-versus-host disease | 2 | 49 | 1.96 | 0.0043 |
| bta05134 | Legionellosis | 2 | 51 | 1.94 | 0.0045 |
| bta05223 | Non-small cell lung cancer | 2 | 60 | 1.87 | 0.0059 |
| bta04151 | PI3K-Akt signaling pathway | 3 | 331 | 1.3 | 0.006 |
| bta04657 | IL-17 signaling pathway | 2 | 83 | 1.73 | 0.0102 |
| bta04640 | Hematopoietic cell lineage | 2 | 92 | 1.68 | 0.0121 |
| bta04658 | Th1 and Th2 cell differentiation | 2 | 92 | 1.68 | 0.0121 |
| bta04620 | Toll-like receptor signaling pathway | 2 | 98 | 1.66 | 0.0128 |
| bta04066 | HIF-1 signaling pathway | 2 | 102 | 1.64 | 0.0134 |
| bta04931 | Insulin resistance | 2 | 106 | 1.62 | 0.0137 |
| bta05142 | Chagas disease | 2 | 106 | 1.62 | 0.0137 |
| bta05146 | Amoebiasis | 2 | 105 | 1.63 | 0.0137 |
| bta04068 | FoxO signaling pathway | 2 | 118 | 1.58 | 0.0158 |
| bta04072 | Phospholipase D signaling pathway | 2 | 138 | 1.51 | 0.0208 |
| bta04217 | Necroptosis | 2 | 144 | 1.49 | 0.022 |
| bta04621 | NOD-like receptor signaling pathway | 2 | 163 | 1.44 | 0.0273 |
| bta05152 | Tuberculosis | 2 | 171 | 1.41 | 0.0292 |
| bta05203 | Viral carcinogenesis | 2 | 193 | 1.36 | 0.036 |
| bta05132 | Salmonella infection | 2 | 198 | 1.35 | 0.037 |

**Table S23:** KEGG pathways that were significantly enriched for differentially expressed genes involved in the interleukin signaling pathway identified from the contrast of wildtype MAC-T cells infected with *Mycobacterium avium* subsp. *Paratuberculosis* (WT-MAP) vs. the *IL10Rα*-knockout MAC-T cells infected with *Mycobacterium avium* subsp. *Paratuberculosis* (KO-MAP)

| **Term ID** | **Term Description** | **Observed Gene Count** | **Background Gene Count** | **Strength** | **False discovery rate** |
| --- | --- | --- | --- | --- | --- |
| bta04061 | Viral protein interaction with cytokine and cytokine receptor | 5 | 83 | 2.04 | 3.02E-07 |
| bta04060 | Cytokine-cytokine receptor interaction | 6 | 283 | 1.59 | 8.52E-07 |
| bta05162 | Measles | 5 | 135 | 1.83 | 1.06E-06 |
| bta05161 | Hepatitis B | 5 | 158 | 1.76 | 1.70E-06 |
| bta04630 | JAK-STAT signaling pathway | 5 | 176 | 1.71 | 2.30E-06 |
| bta05133 | Pertussis | 4 | 70 | 2.02 | 4.22E-06 |
| bta05163 | Human cytomegalovirus infection | 5 | 214 | 1.63 | 4.27E-06 |
| bta05200 | Pathways in cancer | 6 | 488 | 1.35 | 5.20E-06 |
| bta04933 | AGE-RAGE signaling pathway in diabetic complications | 4 | 92 | 1.9 | 7.75E-06 |
| bta05323 | Rheumatoid arthritis | 4 | 91 | 1.9 | 7.75E-06 |
| bta04659 | Th17 cell differentiation | 4 | 107 | 1.83 | 1.19E-05 |
| bta05135 | Yersinia infection | 4 | 124 | 1.77 | 1.93E-05 |
| bta04932 | Non-alcoholic fatty liver disease | 4 | 145 | 1.7 | 3.27E-05 |
| bta04062 | Chemokine signaling pathway | 4 | 170 | 1.63 | 5.64E-05 |
| bta05167 | Kaposi sarcoma-associated herpesvirus infection | 4 | 187 | 1.59 | 7.64E-05 |
| bta05166 | Human T-cell leukemia virus 1 infection | 4 | 213 | 1.53 | 0.00012 |
| bta05321 | Inflammatory bowel disease | 3 | 67 | 1.91 | 0.00018 |
| bta04657 | IL-17 signaling pathway | 3 | 83 | 1.82 | 0.00031 |
| bta04620 | Toll-like receptor signaling pathway | 3 | 98 | 1.75 | 0.00048 |
| bta04931 | Insulin resistance | 3 | 106 | 1.71 | 0.00057 |
| bta05142 | Chagas disease | 3 | 106 | 1.71 | 0.00057 |
| bta04380 | Osteoclast differentiation | 3 | 113 | 1.68 | 0.00063 |
| bta04217 | Necroptosis | 3 | 144 | 1.58 | 0.0012 |
| bta04218 | Cellular senescence | 3 | 148 | 1.57 | 0.0013 |
| bta05164 | Influenza A | 3 | 160 | 1.53 | 0.0015 |
| bta05202 | Transcriptional misregulation in cancer | 3 | 166 | 1.52 | 0.0016 |
| bta05152 | Tuberculosis | 3 | 171 | 1.5 | 0.0017 |
| bta05132 | Salmonella infection | 3 | 198 | 1.44 | 0.0025 |
| bta05169 | Epstein-Barr virus infection | 3 | 201 | 1.43 | 0.0025 |
| bta05144 | Malaria | 2 | 48 | 1.88 | 0.005 |
| bta05332 | Graft-versus-host disease | 2 | 49 | 1.87 | 0.0051 |
| bta04010 | MAPK signaling pathway | 3 | 266 | 1.31 | 0.0052 |
| bta05134 | Legionellosis | 2 | 51 | 1.85 | 0.0052 |
| bta05223 | Non-small cell lung cancer | 2 | 60 | 1.78 | 0.0068 |
| bta05221 | Acute myeloid leukemia | 2 | 62 | 1.77 | 0.0071 |
| bta01521 | EGFR tyrosine kinase inhibitor resistance | 2 | 71 | 1.71 | 0.0089 |
| bta05140 | Leishmaniasis | 2 | 72 | 1.7 | 0.0089 |
| bta04917 | Prolactin signaling pathway | 2 | 74 | 1.69 | 0.0092 |
| bta05235 | PD-L1 expression and PD-1 checkpoint pathway in cancer | 2 | 87 | 1.62 | 0.0122 |
| bta04640 | Hematopoietic cell lineage | 2 | 92 | 1.6 | 0.0133 |
| bta04658 | Th1 and Th2 cell differentiation | 2 | 92 | 1.6 | 0.0133 |
| bta04066 | HIF-1 signaling pathway | 2 | 102 | 1.55 | 0.0154 |
| bta05145 | Toxoplasmosis | 2 | 102 | 1.55 | 0.0154 |
| bta04935 | Growth hormone synthesis, secretion and action | 2 | 106 | 1.54 | 0.0156 |
| bta05146 | Amoebiasis | 2 | 105 | 1.54 | 0.0156 |
| bta04668 | TNF signaling pathway | 2 | 108 | 1.53 | 0.0157 |
| bta04068 | FoxO signaling pathway | 2 | 118 | 1.49 | 0.0183 |
| bta05418 | Fluid shear stress and atherosclerosis | 2 | 128 | 1.45 | 0.021 |
| bta04550 | Signaling pathways regulating pluripotency of stem cells | 2 | 132 | 1.44 | 0.0218 |
| bta04072 | Phospholipase D signaling pathway | 2 | 138 | 1.42 | 0.0233 |
| bta04621 | NOD-like receptor signaling pathway | 2 | 163 | 1.35 | 0.0315 |
| bta05203 | Viral carcinogenesis | 2 | 193 | 1.27 | 0.0428 |

**Table S24:** KEGG pathways that were significantly enriched for differentially expressed genes involved in the interleukin signaling pathway identified from the contrast of the *IL10Rα*-knockout MAC-T cells (KO) vs. the *IL10Rα*-knockout MAC-T cells infected with *Mycobacterium avium* subsp. *Paratuberculosis* (KO-MAP)

| **Term ID** | **Term description** | **Observed Gene Count** | **Background Gene Count** | **Strength** | **False Discovery Rate** |
| --- | --- | --- | --- | --- | --- |
| bta05321 | Inflammatory bowel disease | 2 | 67 | 2.47 | 0.0053 |
| bta04630 | JAK-STAT signaling pathway | 2 | 176 | 2.06 | 0.0179 |
| bta04060 | Cytokine-cytokine receptor interaction | 2 | 283 | 1.85 | 0.0306 |
