# Supplementary material for: The role of interleukin-10 receptor alpha (IL10Rα) in Mycobacterium avium subsp. paratuberculosis infection of a mammary epithelial cell line
Source: BMC Genom Data. 2024 Jun 12;25:58. doi: 10.1186/s12863-024-01234-w (PMC11167801; doi:10.1186/s12863-024-01234-w)
Supplement: Supplementary file 4 — Supplementary Material 4 [file 12863_2024_1234_MOESM4_ESM.docx]

**Table S13:** KEGG pathways that were significantly enriched for differentially expressed genes involved in immune system processes identified from the contrast of wildtype MAC-T cells (WT) vs. the wildtype MAC-T cells infected with *Mycobacterium avium* subsp. *Paratuberculosis* (WT-MAP)

| **Term ID** | **Term Description** | **Observed Gene Count** | **Background Gene Count** | **Strength** | **False Discovery Rate** |
| --- | --- | --- | --- | --- | --- |
| bta04061 | Viral protein interaction with cytokine and cytokine receptor | 14 | 83 | 1.8 | 1.83E-18 |
| bta04060 | Cytokine-cytokine receptor interaction | 16 | 283 | 1.32 | 1.16E-14 |
| bta04062 | Chemokine signaling pathway | 13 | 170 | 1.45 | 2.41E-13 |
| bta04672 | Intestinal immune network for IgA production | 4 | 50 | 1.47 | 0.0015 |
| bta05169 | Epstein-Barr virus infection | 5 | 201 | 0.96 | 0.0208 |
| bta05162 | Measles | 4 | 135 | 1.04 | 0.0405 |

**Table S14:** KEGG pathways that were significantly enriched for differentially expressed genes involved in immune system processes identified from the contrast of wildtype MAC-T cells (WT) vs. the *IL10Rα*-knockout MAC-T cells (KO)

| **Term ID** | **Term Description** | **Observed Gene Count** | **Background Gene Count** | **Strength** | **False Discovery Rate** |
| --- | --- | --- | --- | --- | --- |
| bta04060 | Cytokine-cytokine receptor interaction | 15 | 283 | 1.31 | 3.35E-13 |
| bta04061 | Viral protein interaction with cytokine and cytokine receptor | 10 | 83 | 1.67 | 8.60E-12 |
| bta04062 | Chemokine signaling pathway | 10 | 170 | 1.35 | 4.58E-09 |
| bta04640 | Hematopoietic cell lineage | 5 | 92 | 1.32 | 0.00059 |
| bta04064 | NF-kappa B signaling pathway | 4 | 101 | 1.18 | 0.0144 |
| bta04380 | Osteoclast differentiation | 4 | 113 | 1.13 | 0.0182 |
| bta05144 | Malaria | 3 | 48 | 1.38 | 0.0204 |
| bta04514 | Cell adhesion molecules | 4 | 140 | 1.04 | 0.03 |
| bta05164 | Influenza A | 4 | 160 | 0.98 | 0.0435 |
| bta00590 | Arachidonic acid metabolism | 3 | 73 | 1.2 | 0.0447 |
| bta05202 | Transcriptional misregulation in cancer | 4 | 166 | 0.97 | 0.0447 |

**Table S15:** KEGG pathways that were significantly enriched for differentially expressed genes involved in immune system processes identified from the contrast of wildtype MAC-T cells infected with *Mycobacterium avium* subsp. *Paratuberculosis* (WT-MAP) vs. the *IL10Rα*-knockout MAC-T cells infected with *Mycobacterium avium* subsp. *Paratuberculosis* (KO-MAP)

| **Term ID** | **Term Description** | **Observed Gene Count** | **Background Gene Count** | **Strength** | **False Discovery Rate** |
| --- | --- | --- | --- | --- | --- |
| bta04060 | Cytokine-cytokine receptor interaction | 15 | 283 | 1.28 | 1.15E-12 |
| bta04061 | Viral protein interaction with cytokine and cytokine receptor | 10 | 83 | 1.63 | 1.90E-11 |
| bta04062 | Chemokine signaling pathway | 11 | 170 | 1.36 | 3.79E-10 |
| bta04640 | Hematopoietic cell lineage | 6 | 92 | 1.37 | 3.54E-05 |
| bta05323 | Rheumatoid arthritis | 5 | 91 | 1.29 | 0.00065 |
| bta05142 | Chagas disease | 5 | 106 | 1.23 | 0.0011 |
| bta05163 | Human cytomegalovirus infection | 6 | 214 | 1 | 0.0022 |
| bta04514 | Cell adhesion molecules | 5 | 140 | 1.11 | 0.003 |
| bta04620 | Toll-like receptor signaling pathway | 4 | 98 | 1.16 | 0.0095 |
| bta05144 | Malaria | 3 | 48 | 1.35 | 0.0177 |
| bta04672 | Intestinal immune network for IgA production | 3 | 50 | 1.33 | 0.0181 |
| bta05164 | Influenza A | 4 | 160 | 0.95 | 0.0431 |
| bta05202 | Transcriptional misregulation in cancer | 4 | 166 | 0.93 | 0.0454 |

**Table S16:** Differentially expressed genes related to immune system processes identified from the contrast of the *IL10Rα*-knockout MAC-T cells (KO) vs. the *IL10Rα*-knockout MAC-T cells infected with *Mycobacterium avium* subsp. *Paratuberculosis* (KO-MAP)

| \| **Gene** \| **Regulation** \| **Fold change** \| **Gene description** \| \| --- \| --- \| --- \| --- \| \| OAS1X \| upregulated \| 24.28000652 \| 2'-5' oligoadenylate synthase;OAS1X;ortholog \| \| CCL20 \| upregulated \| 17.75903495 \| C-C motif chemokine 20;CCL20;ortholog \| \| OAS1Y \| upregulated \| 11.14263908 \| 2'-5' oligoadenylate synthase;OAS1Y;ortholog \| \| SEMA7A \| upregulated \| 2.818979594 \| Semaphorin 7A;SEMA7A;ortholog \| \| UBASH3A \| upregulated \| 2.533866071 \| Ubiquitin associated and SH3 domain containing A;UBASH3A;ortholog \| \| ADAM8 \| upregulated \| 2.129546543 \| ADAM metallopeptidase domain 8;ADAM8;ortholog \| \| PTK6 \| upregulated \| 2.070877175 \| Tyrosine-protein kinase;PTK6;ortholog \| \| ISG20 \| upregulated \| 2.020505801 \| Exonuclease domain-containing protein;ISG20;ortholog \| \| NCR3 \| upregulated \| 2.008762696 \| Natural cytotoxicity triggering receptor 3;NCR3;ortholog \| \| MZB1 \| downregulated \| -2.571369626 \| Marginal zone B- and B1-cell-specific protein;MZB1;ortholog \| \| CCL27 \| downregulated \| -2.897522839 \| SCY domain-containing protein;CCL27;ortholog \| \| IFNAG \| downregulated \| -3.345566421 \| Interferon alpha-G;IFNAG;ortholog \| \| CTSL \| downregulated \| -3.792859015 \| Cathepsin L1;CTSL;ortholog \| \| CD74 \| downregulated \| -6.075836193 \| Thyroglobulin type-1 domain-containing protein;CD74;ortholog \| \| TRIM40 \| downregulated \| -7.351168538 \| Tripartite motif containing 40;TRIM40;ortholog \| \| IL36A \| downregulated \| -9.262762513 \| Interleukin-1;IL36A;ortholog \| \| REG3G \| downregulated \| -9.768895627 \| C-type lectin domain-containing protein;REG3G;ortholog \| \| BTNL2 \| downregulated \| -10.09358736 \| BTNL2 protein;BTNL2;ortholog \| \| MPTX \| downregulated \| -12.36606439 \| Mucosal pentraxin;MPTX;ortholog \| |  |  |  |  |
| --- | --- | --- | --- | --- | --- | --- | --- | --- | --- | --- | --- | --- | --- | --- | --- | --- | --- | --- | --- | --- | --- | --- | --- | --- | --- | --- | --- | --- | --- | --- | --- | --- | --- | --- | --- | --- | --- | --- | --- | --- | --- | --- | --- | --- | --- | --- | --- | --- | --- | --- | --- | --- | --- | --- | --- | --- | --- | --- | --- | --- | --- | --- | --- | --- | --- | --- | --- | --- | --- | --- | --- | --- | --- | --- | --- | --- | --- | --- | --- | --- | --- | --- | --- | --- |
