# Supplementary material for: The role of interleukin-10 receptor alpha (IL10Rα) in Mycobacterium avium subsp. paratuberculosis infection of a mammary epithelial cell line
Source: BMC Genom Data. 2024 Jun 12;25:58. doi: 10.1186/s12863-024-01234-w (PMC11167801; doi:10.1186/s12863-024-01234-w)
Supplement: Supplementary file 6 — Supplementary Material 6 [file 12863_2024_1234_MOESM6_ESM.docx]

**Table S25:** KEGG pathways that were significantly enriched for differentially expressed genes involved in involved in the Toll-like receptor signaling pathway identified from the contrast of wildtype MAC-T cells (WT) vs. the wildtype MAC-T cells infected with *Mycobacterium avium* subsp. *Paratuberculosis* (WT-MAP)

| **Term ID** | **Term Description** | **Observed Gene Count** | **Background Gene Count** | **Strength** | **False Discovery Rate** |
| --- | --- | --- | --- | --- | --- |
| bta04620 | Toll-like receptor signaling pathway | 4 | 98 | 2.07 | 1.00E-05 |
| bta04668 | TNF signaling pathway | 4 | 108 | 2.02 | 1.00E-05 |
| bta04657 | IL-17 signaling pathway | 3 | 83 | 2.01 | 0.0004 |
| bta04064 | NF-kappa B signaling pathway | 3 | 101 | 1.93 | 0.00053 |
| bta05132 | Salmonella infection | 3 | 198 | 1.64 | 0.0031 |
| bta04010 | MAPK signaling pathway | 3 | 266 | 1.51 | 0.0061 |
| bta05133 | Pertussis | 2 | 70 | 1.91 | 0.0172 |
| bta05140 | Leishmaniasis | 2 | 72 | 1.9 | 0.0172 |
| bta04625 | C-type lectin receptor signaling pathway | 2 | 94 | 1.78 | 0.0238 |
| bta04660 | T cell receptor signaling pathway | 2 | 98 | 1.77 | 0.0238 |
| bta05142 | Chagas disease | 2 | 106 | 1.73 | 0.0246 |
| bta04921 | Oxytocin signaling pathway | 2 | 136 | 1.62 | 0.0362 |
| bta05162 | Measles | 2 | 135 | 1.63 | 0.0362 |
| bta04145 | Phagosome | 2 | 149 | 1.58 | 0.0376 |
| bta04621 | NOD-like receptor signaling pathway | 2 | 163 | 1.54 | 0.0419 |
| bta05152 | Tuberculosis | 2 | 171 | 1.52 | 0.0431 |
| bta05167 | Kaposi sarcoma-associated herpesvirus infection | 2 | 187 | 1.48 | 0.0483 |
