# Supplementary material for: The role of interleukin-10 receptor alpha (IL10Rα) in Mycobacterium avium subsp. paratuberculosis infection of a mammary epithelial cell line
Source: BMC Genom Data. 2024 Jun 12;25:58. doi: 10.1186/s12863-024-01234-w (PMC11167801; doi:10.1186/s12863-024-01234-w)
Supplement: Supplementary file 7 — Supplementary Material 7 [file 12863_2024_1234_MOESM7_ESM.docx]

**Table S17:** KEGG pathways that were significantly enriched for differentially expressed genes involved in inflammation mediated by the chemokine and cytokine signaling pathway identified from the contrast of wildtype MAC-T cells (WT) vs. the wildtype MAC-T cells infected with *Mycobacterium avium* subsp. *Paratuberculosis* (WT-MAP)

| **Term ID** | **Term Description** | **Observed Gene Count** | **Background Gene Count** | **Strength** | **False Discovery Rate** |
| --- | --- | --- | --- | --- | --- |
| bta04061 | Viral protein interaction with cytokine and cytokine receptor | 11 | 83 | 1.96 | 1.96E-16 |
| bta04062 | Chemokine signaling pathway | 11 | 170 | 1.65 | 1.65E-13 |
| bta04060 | Cytokine-cytokine receptor interaction | 11 | 283 | 1.43 | 2.35E-11 |
| bta04657 | IL-17 signaling pathway | 6 | 83 | 1.7 | 3.23E-07 |
| bta04668 | TNF signaling pathway | 5 | 108 | 1.5 | 5.07E-05 |
| bta04672 | Intestinal immune network for IgA production | 4 | 50 | 1.74 | 8.08E-05 |
| bta05163 | Human cytomegalovirus infection | 5 | 214 | 1.21 | 0.00093 |
| bta05140 | Leishmaniasis | 3 | 72 | 1.46 | 0.009 |
| bta05167 | Kaposi sarcoma-associated herpesvirus infection | 4 | 187 | 1.17 | 0.009 |
| bta04625 | C-type lectin receptor signaling pathway | 3 | 94 | 1.34 | 0.0152 |
| bta05323 | Rheumatoid arthritis | 3 | 91 | 1.36 | 0.0152 |
| bta04620 | Toll-like receptor signaling pathway | 3 | 98 | 1.32 | 0.0157 |
| bta05135 | Yersinia infection | 3 | 124 | 1.22 | 0.0282 |

**Table S18:** KEGG pathways that were significantly enriched for differentially expressed genes involved in inflammation mediated by the chemokine and cytokine signaling pathway identified from the contrast of wildtype MAC-T cells (WT) vs. the *IL10Rα*-knockout MAC-T cells (KO)

| **Term ID** | **Term description** | **Observed Gene Count** | **Background Gene Count** | **Strength** | **False Discovery Rate** |
| --- | --- | --- | --- | --- | --- |
| bta04062 | Chemokine signaling pathway | 12 | 170 | 1.81 | 6.01E-17 |
| bta05163 | Human cytomegalovirus infection | 10 | 214 | 1.63 | 3.29E-12 |
| bta04061 | Viral protein interaction with cytokine and cytokine receptor | 8 | 83 | 1.94 | 6.13E-12 |
| bta04060 | Cytokine-cytokine receptor interaction | 9 | 283 | 1.46 | 1.27E-09 |
| bta04726 | Serotonergic synapse | 7 | 106 | 1.78 | 2.19E-09 |
| bta04657 | IL-17 signaling pathway | 5 | 83 | 1.74 | 2.40E-06 |
| bta05200 | Pathways in cancer | 8 | 488 | 1.17 | 2.40E-06 |
| bta04933 | AGE-RAGE signaling pathway in diabetic complications | 5 | 92 | 1.69 | 3.37E-06 |
| bta05146 | Amoebiasis | 5 | 105 | 1.64 | 5.64E-06 |
| bta04723 | Retrograde endocannabinoid signaling | 5 | 144 | 1.5 | 2.31E-05 |
| bta04713 | Circadian entrainment | 4 | 90 | 1.61 | 0.00013 |
| bta04750 | Inflammatory mediator regulation of TRP channels | 4 | 97 | 1.57 | 0.00016 |
| bta04064 | NF-kappa B signaling pathway | 4 | 101 | 1.56 | 0.00017 |
| bta04724 | Glutamatergic synapse | 4 | 102 | 1.55 | 0.00017 |
| bta04725 | Cholinergic synapse | 4 | 106 | 1.54 | 0.00018 |
| bta04919 | Thyroid hormone signaling pathway | 4 | 110 | 1.52 | 0.00018 |
| bta05142 | Chagas disease | 4 | 106 | 1.54 | 0.00018 |
| bta04728 | Dopaminergic synapse | 4 | 122 | 1.47 | 0.00025 |
| bta05135 | Yersinia infection | 4 | 124 | 1.47 | 0.00025 |
| bta05143 | African trypanosomiasis | 3 | 36 | 1.88 | 0.00025 |
| bta04921 | Oxytocin signaling pathway | 4 | 136 | 1.43 | 0.00033 |
| bta04310 | Wnt signaling pathway | 4 | 146 | 1.4 | 0.00041 |
| bta04370 | VEGF signaling pathway | 3 | 48 | 1.75 | 0.00046 |
| bta04961 | Endocrine and other factor-regulated calcium reabsorption | 3 | 48 | 1.75 | 0.00046 |
| bta05144 | Malaria | 3 | 48 | 1.75 | 0.00046 |
| bta05164 | Influenza A | 4 | 160 | 1.36 | 0.00049 |
| bta04621 | NOD-like receptor signaling pathway | 4 | 163 | 1.35 | 0.00051 |
| bta04923 | Regulation of lipolysis in adipocytes | 3 | 54 | 1.7 | 0.00053 |
| bta04510 | Focal adhesion | 4 | 169 | 1.33 | 0.00055 |
| bta04720 | Long-term potentiation | 3 | 57 | 1.68 | 0.00057 |
| bta05167 | Kaposi sarcoma-associated herpesvirus infection | 4 | 187 | 1.29 | 0.00075 |
| bta04971 | Gastric acid secretion | 3 | 70 | 1.59 | 0.00097 |
| bta04918 | Thyroid hormone synthesis | 3 | 71 | 1.58 | 0.00098 |
| bta00590 | Arachidonic acid metabolism | 3 | 73 | 1.57 | 0.001 |
| bta04014 | Ras signaling pathway | 4 | 218 | 1.22 | 0.0012 |
| bta04540 | Gap junction | 3 | 81 | 1.53 | 0.0012 |
| bta04911 | Insulin secretion | 3 | 79 | 1.54 | 0.0012 |
| bta04912 | GnRH signaling pathway | 3 | 80 | 1.53 | 0.0012 |
| bta04727 | GABAergic synapse | 3 | 84 | 1.51 | 0.0013 |
| bta04925 | Aldosterone synthesis and secretion | 3 | 88 | 1.49 | 0.0015 |
| bta05032 | Morphine addiction | 3 | 89 | 1.49 | 0.0015 |
| bta04916 | Melanogenesis | 3 | 93 | 1.47 | 0.0016 |
| bta04970 | Salivary secretion | 3 | 92 | 1.47 | 0.0016 |
| bta05323 | Rheumatoid arthritis | 3 | 91 | 1.48 | 0.0016 |
| bta04928 | Parathyroid hormone synthesis, secretion and action | 3 | 98 | 1.44 | 0.0018 |
| bta04935 | Growth hormone synthesis, secretion and action | 3 | 106 | 1.41 | 0.0022 |
| bta04668 | TNF signaling pathway | 3 | 108 | 1.4 | 0.0023 |
| bta04611 | Platelet activation | 3 | 112 | 1.39 | 0.0025 |
| bta04071 | Sphingolipid signaling pathway | 3 | 115 | 1.37 | 0.0026 |
| bta04926 | Relaxin signaling pathway | 3 | 121 | 1.35 | 0.0029 |
| bta05165 | Human papillomavirus infection | 4 | 303 | 1.08 | 0.0029 |
| bta04270 | Vascular smooth muscle contraction | 3 | 123 | 1.35 | 0.003 |
| bta04371 | Apelin signaling pathway | 3 | 129 | 1.32 | 0.0034 |
| bta04151 | PI3K-Akt signaling pathway | 4 | 331 | 1.04 | 0.0037 |
| bta04072 | Phospholipase D signaling pathway | 3 | 138 | 1.3 | 0.0039 |
| bta04261 | Adrenergic signaling in cardiomyocytes | 3 | 138 | 1.3 | 0.0039 |
| bta05161 | Hepatitis B | 3 | 158 | 1.24 | 0.0056 |
| bta01523 | Antifolate resistance | 2 | 38 | 1.68 | 0.0069 |
| bta04973 | Carbohydrate digestion and absorption | 2 | 40 | 1.66 | 0.0074 |
| bta04020 | Calcium signaling pathway | 3 | 187 | 1.16 | 0.0086 |
| bta04015 | Rap1 signaling pathway | 3 | 191 | 1.15 | 0.009 |
| bta05134 | Legionellosis | 2 | 51 | 1.55 | 0.0111 |
| bta05170 | Human immunodeficiency virus 1 infection | 3 | 207 | 1.12 | 0.0111 |
| bta04730 | Long-term depression | 2 | 53 | 1.54 | 0.0117 |
| bta04913 | Ovarian steroidogenesis | 2 | 54 | 1.53 | 0.012 |
| bta05031 | Amphetamine addiction | 2 | 59 | 1.49 | 0.014 |
| bta04927 | Cortisol synthesis and secretion | 2 | 60 | 1.48 | 0.0142 |
| bta04929 | GnRH secretion | 2 | 60 | 1.48 | 0.0142 |
| bta04664 | Fc epsilon RI signaling pathway | 2 | 63 | 1.46 | 0.0151 |
| bta05416 | Viral myocarditis | 2 | 63 | 1.46 | 0.0151 |
| bta04662 | B cell receptor signaling pathway | 2 | 65 | 1.45 | 0.0156 |
| bta04742 | Taste transduction | 2 | 66 | 1.44 | 0.0156 |
| bta04924 | Renin secretion | 2 | 65 | 1.45 | 0.0156 |
| bta05020 | Prion disease | 3 | 255 | 1.03 | 0.0167 |
| bta05133 | Pertussis | 2 | 70 | 1.41 | 0.017 |
| bta01521 | EGFR tyrosine kinase inhibitor resistance | 2 | 71 | 1.41 | 0.0173 |
| bta05140 | Leishmaniasis | 2 | 72 | 1.4 | 0.0175 |
| bta04010 | MAPK signaling pathway | 3 | 266 | 1.01 | 0.0178 |
| bta04512 | ECM-receptor interaction | 2 | 77 | 1.37 | 0.0194 |
| bta04666 | Fc gamma R-mediated phagocytosis | 2 | 79 | 1.36 | 0.0201 |
| bta05410 | Hypertrophic cardiomyopathy | 2 | 84 | 1.34 | 0.0223 |
| bta04070 | Phosphatidylinositol signaling system | 2 | 89 | 1.31 | 0.0246 |
| bta05231 | Choline metabolism in cancer | 2 | 89 | 1.31 | 0.0246 |
| bta05414 | Dilated cardiomyopathy | 2 | 89 | 1.31 | 0.0246 |
| bta04922 | Glucagon signaling pathway | 2 | 93 | 1.29 | 0.0259 |
| bta04625 | C-type lectin receptor signaling pathway | 2 | 94 | 1.29 | 0.0261 |
| bta04972 | Pancreatic secretion | 2 | 95 | 1.28 | 0.0263 |
| bta04620 | Toll-like receptor signaling pathway | 2 | 98 | 1.27 | 0.0276 |
| bta04066 | HIF-1 signaling pathway | 2 | 102 | 1.25 | 0.0294 |
| bta04670 | Leukocyte transendothelial migration | 2 | 105 | 1.24 | 0.0308 |
| bta04931 | Insulin resistance | 2 | 106 | 1.23 | 0.031 |
| bta05010 | Alzheimer disease | 3 | 347 | 0.9 | 0.0315 |
| bta04974 | Protein digestion and absorption | 2 | 112 | 1.21 | 0.0337 |
| bta04650 | Natural killer cell mediated cytotoxicity | 2 | 115 | 1.2 | 0.035 |
| bta04915 | Estrogen signaling pathway | 2 | 119 | 1.18 | 0.037 |
| bta05418 | Fluid shear stress and atherosclerosis | 2 | 128 | 1.15 | 0.042 |
| bta05017 | Spinocerebellar ataxia | 2 | 131 | 1.14 | 0.0434 |
| bta04934 | Cushing syndrome | 2 | 141 | 1.11 | 0.0494 |

**Table S19:** KEGG pathways that were significantly enriched for differentially expressed genes involved in inflammation mediated by the chemokine and cytokine signaling pathway identified from the contrast of wildtype MAC-T cells infected with *Mycobacterium avium* subsp. *Paratuberculosis* (WT-MAP) vs. the *IL10Rα*-knockout MAC-T cells infected with *Mycobacterium avium* subsp. *Paratuberculosis* (KO-MAP)

| **Term ID** | **Term Description** | **Observed Gene Count** | **Background Gene Count** | **Strength** | **False Discovery Rate** | |
| --- | --- | --- | --- | --- | --- | --- |
| bta04062 | Chemokine signaling pathway | 12 | 170 | 1.77 | 2.47E-16 |  |
| bta04061 | Viral protein interaction with cytokine and cytokine receptor | 10 | 83 | 2 | 1.20E-15 |  |
| bta04060 | Cytokine-cytokine receptor interaction | 11 | 283 | 1.51 | 1.82E-12 |  |
| bta05163 | Human cytomegalovirus infection | 10 | 214 | 1.59 | 4.89E-12 |  |
| bta04657 | IL-17 signaling pathway | 5 | 83 | 1.7 | 5.29E-06 |  |
| bta04668 | TNF signaling pathway | 5 | 108 | 1.59 | 1.55E-05 |  |
| bta05200 | Pathways in cancer | 7 | 488 | 1.08 | 8.45E-05 |  |
| bta04933 | AGE-RAGE signaling pathway in diabetic complications | 4 | 92 | 1.56 | 0.00027 |  |
| bta05323 | Rheumatoid arthritis | 4 | 91 | 1.56 | 0.00027 |  |
| bta05142 | Chagas disease | 4 | 106 | 1.5 | 0.00038 |  |
| bta05135 | Yersinia infection | 4 | 124 | 1.43 | 0.00063 |  |
| bta05144 | Malaria | 3 | 48 | 1.72 | 0.0011 |  |
| bta05165 | Human papillomavirus infection | 5 | 303 | 1.14 | 0.0011 |  |
| bta04621 | NOD-like receptor signaling pathway | 4 | 163 | 1.31 | 0.0013 |  |
| bta05164 | Influenza A | 4 | 160 | 1.32 | 0.0013 |  |
| bta04510 | Focal adhesion | 4 | 169 | 1.29 | 0.0014 |  |
| bta05167 | Kaposi sarcoma-associated herpesvirus infection | 4 | 187 | 1.25 | 0.002 |  |
| bta04512 | ECM-receptor interaction | 3 | 77 | 1.51 | 0.003 |  |
| bta04064 | NF-kappa B signaling pathway | 3 | 101 | 1.39 | 0.0056 |  |
| bta04620 | Toll-like receptor signaling pathway | 3 | 98 | 1.41 | 0.0056 |  |
| bta05020 | Prion disease | 4 | 255 | 1.12 | 0.0056 |  |
| bta05146 | Amoebiasis | 3 | 105 | 1.38 | 0.0059 |  |
| bta05168 | Herpes simplex virus 1 infection | 4 | 289 | 1.06 | 0.0075 |  |
| bta04151 | PI3K-Akt signaling pathway | 4 | 331 | 1 | 0.0119 |  |
| bta05161 | Hepatitis B | 3 | 158 | 1.2 | 0.0166 |  |
| bta04020 | Calcium signaling pathway | 3 | 187 | 1.13 | 0.0257 |  |
| bta04370 | VEGF signaling pathway | 2 | 48 | 1.54 | 0.0274 |  |
| bta04672 | Intestinal immune network for IgA production | 2 | 50 | 1.52 | 0.0286 |  |
| bta05134 | Legionellosis | 2 | 51 | 1.51 | 0.0286 |  |
| bta04913 | Ovarian steroidogenesis | 2 | 54 | 1.49 | 0.0309 |  |
| bta04923 | Regulation of lipolysis in adipocytes | 2 | 54 | 1.49 | 0.0309 |  |
| bta05166 | Human T-cell leukemia virus 1 infection | 3 | 213 | 1.07 | 0.0309 |  |
| bta04623 | Cytosolic DNA-sensing pathway | 2 | 60 | 1.44 | 0.0343 |  |
| bta05212 | Pancreatic cancer | 2 | 67 | 1.4 | 0.0411 |  |
| bta05321 | Inflammatory bowel disease | 2 | 67 | 1.4 | 0.0411 |  |
| bta01521 | EGFR tyrosine kinase inhibitor resistance | 2 | 71 | 1.37 | 0.0422 |  |
| bta05133 | Pertussis | 2 | 70 | 1.38 | 0.0422 |  |
| bta04610 | Complement and coagulation cascades | 2 | 78 | 1.33 | 0.0491 |  |

**Table S20:** KEGG pathways that were significantly enriched for differentially expressed genes involved in inflammation mediated by the chemokine and cytokine signaling pathway identified from the contrast of the *IL10Rα*-knockout MAC-T cells (KO) vs. the *IL10Rα*-knockout MAC-T cells infected with *Mycobacterium avium* subsp. *Paratuberculosis* (KO-MAP)

| **Term ID** | **Term Description** | **Observed Gene Count** | **Background Gene Count** | **Strength** | **False Discovery Rate** |
| --- | --- | --- | --- | --- | --- |
| bta04062 | Chemokine signaling pathway | 5 | 170 | 1.82 | 2.69E-06 |
| bta05143 | African trypanosomiasis | 3 | 36 | 2.27 | 0.00013 |
| bta05163 | Human cytomegalovirus infection | 4 | 214 | 1.62 | 0.00025 |
| bta04060 | Cytokine-cytokine receptor interaction | 4 | 283 | 1.5 | 0.00044 |
| bta04061 | Viral protein interaction with cytokine and cytokine receptor | 3 | 83 | 1.9 | 0.00044 |
| bta04270 | Vascular smooth muscle contraction | 3 | 123 | 1.73 | 0.00044 |
| bta04540 | Gap junction | 3 | 81 | 1.92 | 0.00044 |
| bta04713 | Circadian entrainment | 3 | 90 | 1.87 | 0.00044 |
| bta04724 | Glutamatergic synapse | 3 | 102 | 1.82 | 0.00044 |
| bta04725 | Cholinergic synapse | 3 | 106 | 1.8 | 0.00044 |
| bta04726 | Serotonergic synapse | 3 | 106 | 1.8 | 0.00044 |
| bta04728 | Dopaminergic synapse | 3 | 122 | 1.74 | 0.00044 |
| bta04750 | Inflammatory mediator regulation of TRP channels | 3 | 97 | 1.84 | 0.00044 |
| bta04911 | Insulin secretion | 3 | 79 | 1.93 | 0.00044 |
| bta04912 | GnRH signaling pathway | 3 | 80 | 1.92 | 0.00044 |
| bta04916 | Melanogenesis | 3 | 93 | 1.86 | 0.00044 |
| bta04918 | Thyroid hormone synthesis | 3 | 71 | 1.97 | 0.00044 |
| bta04925 | Aldosterone synthesis and secretion | 3 | 88 | 1.88 | 0.00044 |
| bta04928 | Parathyroid hormone synthesis, secretion and action | 3 | 98 | 1.83 | 0.00044 |
| bta04933 | AGE-RAGE signaling pathway in diabetic complications | 3 | 92 | 1.86 | 0.00044 |
| bta04935 | Growth hormone synthesis, secretion and action | 3 | 106 | 1.8 | 0.00044 |
| bta04970 | Salivary secretion | 3 | 92 | 1.86 | 0.00044 |
| bta04971 | Gastric acid secretion | 3 | 70 | 1.98 | 0.00044 |
| bta04972 | Pancreatic secretion | 3 | 95 | 1.85 | 0.00044 |
| bta05146 | Amoebiasis | 3 | 105 | 1.8 | 0.00044 |
| bta04921 | Oxytocin signaling pathway | 3 | 136 | 1.69 | 0.00047 |
| bta04723 | Retrograde endocannabinoid signaling | 3 | 144 | 1.67 | 0.00053 |
| bta05200 | Pathways in cancer | 4 | 488 | 1.26 | 0.00067 |
| bta04015 | Rap1 signaling pathway | 3 | 191 | 1.54 | 0.0011 |
| bta04973 | Carbohydrate digestion and absorption | 2 | 40 | 2.05 | 0.0023 |
| bta04961 | Endocrine and other factor-regulated calcium reabsorption | 2 | 48 | 1.97 | 0.0032 |
| bta04730 | Long-term depression | 2 | 53 | 1.92 | 0.0037 |
| bta04720 | Long-term potentiation | 2 | 57 | 1.89 | 0.0042 |
| bta05031 | Amphetamine addiction | 2 | 59 | 1.88 | 0.0043 |
| bta04927 | Cortisol synthesis and secretion | 2 | 60 | 1.87 | 0.0044 |
| bta04929 | GnRH secretion | 2 | 60 | 1.87 | 0.0044 |
| bta04924 | Renin secretion | 2 | 65 | 1.83 | 0.0048 |
| bta01521 | EGFR tyrosine kinase inhibitor resistance | 2 | 71 | 1.8 | 0.0056 |
| bta04657 | IL-17 signaling pathway | 2 | 83 | 1.73 | 0.0073 |
| bta04727 | GABAergic synapse | 2 | 84 | 1.72 | 0.0073 |
| bta04070 | Phosphatidylinositol signaling system | 2 | 89 | 1.7 | 0.008 |
| bta05032 | Morphine addiction | 2 | 89 | 1.7 | 0.008 |
| bta05323 | Rheumatoid arthritis | 2 | 91 | 1.69 | 0.008 |
| bta04066 | HIF-1 signaling pathway | 2 | 102 | 1.64 | 0.0097 |
| bta04668 | TNF signaling pathway | 2 | 108 | 1.61 | 0.0102 |
| bta04931 | Insulin resistance | 2 | 106 | 1.62 | 0.0102 |
| bta05142 | Chagas disease | 2 | 106 | 1.62 | 0.0102 |
| bta04919 | Thyroid hormone signaling pathway | 2 | 110 | 1.61 | 0.0103 |
| bta04611 | Platelet activation | 2 | 112 | 1.6 | 0.0105 |
| bta04071 | Sphingolipid signaling pathway | 2 | 115 | 1.59 | 0.0108 |
| bta04915 | Estrogen signaling pathway | 2 | 119 | 1.57 | 0.0113 |
| bta04926 | Relaxin signaling pathway | 2 | 121 | 1.56 | 0.0115 |
| bta04371 | Apelin signaling pathway | 2 | 129 | 1.54 | 0.0127 |
| bta05017 | Spinocerebellar ataxia | 2 | 131 | 1.53 | 0.0129 |
| bta04072 | Phospholipase D signaling pathway | 2 | 138 | 1.51 | 0.014 |
| bta04261 | Adrenergic signaling in cardiomyocytes | 2 | 138 | 1.51 | 0.014 |
| bta04934 | Cushing syndrome | 2 | 141 | 1.5 | 0.0141 |
| bta04310 | Wnt signaling pathway | 2 | 146 | 1.48 | 0.0148 |
| bta04022 | cGMP-PKG signaling pathway | 2 | 157 | 1.45 | 0.0168 |
| bta05161 | Hepatitis B | 2 | 158 | 1.45 | 0.0168 |
| bta05164 | Influenza A | 2 | 160 | 1.44 | 0.0168 |
| bta04621 | NOD-like receptor signaling pathway | 2 | 163 | 1.44 | 0.0172 |
| bta04020 | Calcium signaling pathway | 2 | 187 | 1.38 | 0.0221 |
| bta05166 | Human T-cell leukemia virus 1 infection | 2 | 213 | 1.32 | 0.0279 |
