# Supplementary material for: The role of interleukin-10 receptor alpha (IL10Rα) in Mycobacterium avium subsp. paratuberculosis infection of a mammary epithelial cell line
Source: BMC Genom Data. 2024 Jun 12;25:58. doi: 10.1186/s12863-024-01234-w (PMC11167801; doi:10.1186/s12863-024-01234-w)
Supplement: Supplementary file 8 — Supplementary Material 8 [file 12863_2024_1234_MOESM8_ESM.docx]

**Table S29:** Differentially expressed genes related to the B cell activation pathway were identified from the contrast of wildtype MAC-T cells (WT) vs. the wildtype MAC-T cells infected with *Mycobacterium avium* subsp. *Paratuberculosis* (WT-MAP)

| **Gene** | **Regulation** | **Fold change** | **Gene description** |
| --- | --- | --- | --- |
| GRAP | upregulated | -33.455121 | GRB2-related adapter protein;GRAP;ortholog |
| BLK | upregulated | -22.53966 | Tyrosine-protein kinase;BLK;ortholog |
| BTK | upregulated | -22.47256 | Tyrosine-protein kinase;BTK;ortholog |
| PTPRC | upregulated | -20.882156 | Protein-tyrosine-phosphatase;PTPRC;ortholog |
| CD79A | upregulated | -9.0294804 | B-cell antigen receptor complex-associated protein alpha chain;CD79A;ortholog |
| MAPK12 | upregulated | -2.9233588 | Protein kinase domain-containing protein;MAPK12;ortholog |
| JUN | upregulated | -2.0403603 | Activator protein 1;JUN;ortholog |
| FOS | downregulated | 2.05376215 | Proto-oncogene c-Fos;FOS;ortholog |

**Table S30:** Differentially expressed genes related to the T cell activation pathway were identified from the contrast of wildtype MAC-T cells (WT) vs. the wildtype MAC-T cells infected with *Mycobacterium avium* subsp. *Paratuberculosis* (WT-MAP)

| **Gene** | **Regulation** | **Fold change** | **Gene description** |
| --- | --- | --- | --- |
| BOLA-DQA5 | upregulated | -35.866098 | Ig-like domain-containing protein;BOLA-DQA5;ortholog |
| PTPRC | upregulated | -20.882156 | Protein-tyrosine-phosphatase;PTPRC;ortholog |
| CD74 | upregulated | -20.180792 | Thyroglobulin type-1 domain-containing protein;CD74;ortholog |
| BOLA-DRA | upregulated | -17.948689 | Ig-like domain-containing protein;BoLA-DRA;ortholog |
| CD3G | upregulated | -4.878869 | T-cell surface glycoprotein CD3 gamma chain;CD3G;ortholog |
| JUN | upregulated | -2.0403603 | Activator protein 1;JUN;ortholog |
| CD80 | downregulated | 2.0151518 | CD80 molecule;CD80;ortholog |
| FOS | downregulated | 2.05376215 | Proto-oncogene c-Fos;FOS;ortholog |

**Table S31:** Differentially expressed genes related to the B cell activation pathway were identified from the contrast of wildtype MAC-T cells (WT) vs. the *IL10Rα*-knockout MAC-T cells (KO)

| **Gene** | **Regulation** | **Fold change** | **Gene description** |
| --- | --- | --- | --- |
| PRKCB | upregulated | 4.89489978 | Protein kinase C beta type;PRKCB;ortholog |
| MAPK13 | downregulated | -2.0410443 | Mitogen-activated protein kinase 13;MAPK13;ortholog |
| MAPK12 | downregulated | -2.1202796 | Protein kinase domain-containing protein;MAPK12;ortholog |
| RHOV | downregulated | -2.5284053 | Rho-related GTP-binding protein RhoV;Rhov;ortholog |
| RAC2 | downregulated | -2.8334285 | Ras-related C3 botulinum toxin substrate 2;RAC2;ortholog |
| VAV3 | downregulated | -4.1837696 | Vav guanine nucleotide exchange factor 3;VAV3;ortholog |
| PTPRC | downregulated | -5.3437136 | Protein-tyrosine-phosphatase;PTPRC;ortholog |
| LYN | downregulated | -10.770528 | Tyrosine-protein kinase;LYN;ortholog |
| BLK | downregulated | -23.922812 | Tyrosine-protein kinase;BLK;ortholog |

**Table S32:** Differentially expressed genes related to the T cell activation pathway were identified from the contrast of wildtype MAC-T cells (WT) vs. the *IL10Rα*-knockout MAC-T cells (KO)

| **Gene** | **Regulation** | **Fold change** | **Gene description** |
| --- | --- | --- | --- |
| RAC2 | downregulated | -2.8334285 | Ras-related C3 botulinum toxin substrate 2;RAC2;ortholog |
| VAV3 | downregulated | -4.1837696 | Vav guanine nucleotide exchange factor 3;VAV3;ortholog |
| CD74 | downregulated | -5.2298128 | Thyroglobulin type-1 domain-containing protein;CD74;ortholog |
| PTPRC | downregulated | -5.3437136 | Protein-tyrosine-phosphatase;PTPRC;ortholog |
| BOLA-DRA | downregulated | -8.1594337 | Ig-like domain-containing protein;BoLA-DRA;ortholog |
| CD3D | downregulated | -11.738271 | T-cell surface glycoprotein CD3 delta chain;CD3D;ortholog |

**Table S33:** Differentially expressed genes related to the B cell activation pathway were identified from the contrast of wildtype MAC-T cells infected with *Mycobacterium avium* subsp. *Paratuberculosis* (WT-MAP) vs. the *IL10Rα*-knockout MAC-T cells infected with *Mycobacterium avium* subsp. *Paratuberculosis* (KO-MAP)

| **Gene** | **Regulation** | **Fold change** | **Gene description** |
| --- | --- | --- | --- |
| RHOV | downregulated | -2.6540636 | Rho-related GTP-binding protein RhoV;Rhov;ortholog |
| FOS | downregulated | -3.2168123 | Proto-oncogene c-Fos;FOS;ortholog |
| RAC2 | downregulated | -3.5887588 | Ras-related C3 botulinum toxin substrate 2;RAC2;ortholog |
| VAV3 | downregulated | -4.5160134 | Vav guanine nucleotide exchange factor 3;VAV3;ortholog |
| LYN | downregulated | -10.678549 | Tyrosine-protein kinase;LYN;ortholog |

**Table S34:** Differentially expressed genes related to the T cell activation pathway were identified from the contrast of wildtype MAC-T cells infected with *Mycobacterium avium* subsp. *Paratuberculosis* (WT-MAP) vs. the *IL10Rα*-knockout MAC-T cells infected with *Mycobacterium avium* subsp. *Paratuberculosis* (KO-MAP)

| **Gene** | **Regulation** | **Fold change** | **Gene description** |
| --- | --- | --- | --- |
| CD3G | upregulated | 4.48262094 | T-cell surface glycoprotein CD3 gamma chain;CD3G;ortholog |
| BOLA-DRA | downregulated | -2.8538691 | Ig-like domain-containing protein;BoLA-DRA;ortholog |
| FOS | downregulated | -3.2168123 | Proto-oncogene c-Fos;FOS;ortholog |
| CD80 | downregulated | -3.2772219 | CD80 molecule;CD80;ortholog |
| RAC2 | downregulated | -3.5887588 | Ras-related C3 botulinum toxin substrate 2;RAC2;ortholog |
| VAV3 | downregulated | -4.5160134 | Vav guanine nucleotide exchange factor 3;VAV3;ortholog |
| CD3D | downregulated | -5.2699614 | T-cell surface glycoprotein CD3 delta chain;CD3D;ortholog |

**Table S35:** Differentially expressed genes related to the B cell activation pathway were identified from the contrast of the *IL10Rα*-knockout MAC-T cells (KO) vs. the *IL10Rα*-knockout MAC-T cells infected with *Mycobacterium avium* subsp. *Paratuberculosis* (KO-MAP)

| **Gene** | **Regulation** | **Fold change** | **Gene description** |
| --- | --- | --- | --- |
| PRKCB | downregulated | -3.1386525 | Protein kinase C beta type;PRKCB;ortholog |

**Table S36:** Differentially expressed genes related to the T cell activation pathway were identified from the contrast of the *IL10Rα*-knockout MAC-T cells (KO) vs. the *IL10Rα*-knockout MAC-T cells infected with *Mycobacterium avium* subsp. *Paratuberculosis* (KO-MAP)

| **Gene** | **Regulation** | **Fold change** | **Gene description** |
| --- | --- | --- | --- |
| CD74 | downregulated | -6.0758362 | Thyroglobulin type-1 domain-containing protein;CD74;ortholog |
| BOLA-DRA | downregulated | -6.2422791 | Ig-like domain-containing protein;BoLA-DRA;ortholog |
