# Supplementary material for: The role of interleukin-10 receptor alpha (IL10Rα) in Mycobacterium avium subsp. paratuberculosis infection of a mammary epithelial cell line
Source: BMC Genom Data. 2024 Jun 12;25:58. doi: 10.1186/s12863-024-01234-w (PMC11167801; doi:10.1186/s12863-024-01234-w)
Supplement: Supplementary file 10 — Supplementary Material 10 [file 12863_2024_1234_MOESM10_ESM.docx]

**Table S26:** Differentially expressed genes related to inflammation were identified from the contrast of the *IL10Rα*-knockout MAC-T cells (KO) vs. the *IL10Rα*-knockout MAC-T cells infected with *Mycobacterium avium* subsp. *Paratuberculosis* (KO-MAP)

| **Gene** | **Regulation** | **Fold change** | **Gene description** |
| --- | --- | --- | --- |
| CCL20 | upregulated | 17.759035 | C-C motif chemokine 20;CCL20;ortholog |
| IL6 | upregulated | 2.73266584 | Interleukin-6;IL6;ortholog |
| MYLK | downregulated | -2.3649898 | Myosin light chain kinase, smooth muscle;MYLK;ortholog |
| CCL27 | downregulated | -2.8975228 | SCY domain-containing protein;CCL27;ortholog |
| PRKCB | downregulated | -3.1386525 | Protein kinase C beta type;PRKCB;ortholog |
| ACTG2 | downregulated | -5.0182061 | Actin, gamma-enteric smooth muscle;ACTG2;ortholog |
| ADCY5 | downregulated | -5.0951012 | Adenylate cyclase type 5;ADCY5;ortholog |
| IL36A | downregulated | -9.2627625 | Interleukin-1;IL36A;ortholog |
| PLCB4 | downregulated | -24.941168 | 1-phosphatidylinositol 4,5-bisphosphate phosphodiesterase beta-4;PLCB4;ortholog |

**Table S27:** Differentially expressed genes related to the interleukin signalling pathwasy were identified from the contrast of the *IL10Rα*-knockout MAC-T cells (KO) vs. the *IL10Rα*-knockout MAC-T cells infected with *Mycobacterium avium* subsp. *Paratuberculosis* (KO-MAP)

| **Gene** | **Regulation** | **Fold change** | **Gene description** |
| --- | --- | --- | --- |
| IL6 | upregulated | 2.73266584 | Interleukin-6;IL6;ortholog |
| IL12RB2 | downregulated | -6.7284615 | Interleukin-12 receptor subunit beta-2;IL12RB2;ortholog |
