# Supplementary material for: The role of interleukin-10 receptor alpha (IL10Rα) in Mycobacterium avium subsp. paratuberculosis infection of a mammary epithelial cell line
Source: BMC Genom Data. 2024 Jun 12;25:58. doi: 10.1186/s12863-024-01234-w (PMC11167801; doi:10.1186/s12863-024-01234-w)

**Figure S1:** Venn diagram of number of differentially expressed genes shared and exclusive to the four contrasts: (1) wildtype MAC-T cells (WT) versus the wildtype MAC-T cells infected with *Mycobacterium avium* subsp. *Paratuberculosis* (WT-MAP); (2) wildtype MAC-T cells (WT) versus the *IL10Rα*-knockout MAC-T cells (KO); (3) wildtype MAC-T cells infected with *Mycobacterium avium* subsp. *Paratuberculosis* (WT-MAP) versus the *IL10Rα*-knockout MAC-T cells infected with *Mycobacterium avium* subsp. *Paratuberculosis* (KO-MAP); and (4) *IL10Rα*-knockout MAC-T cells (KO) versus the *IL10Rα*-knockout MAC-T cells infected with *Mycobacterium avium* subsp. *Paratuberculosis* (KO-MAP)


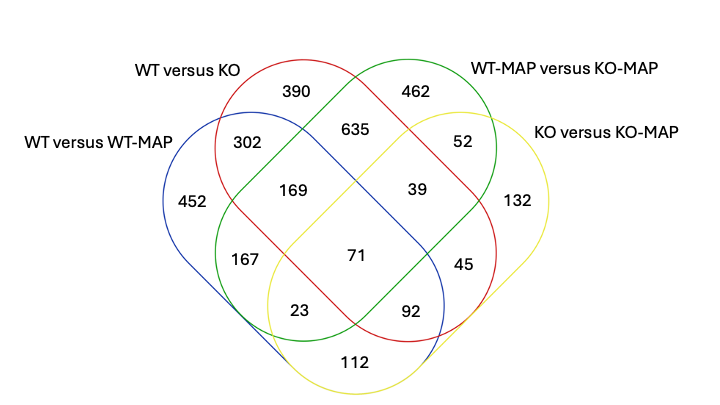

Supplement: Supplementary file 11 — Supplementary Material 11 [file 12863_2024_1234_MOESM11_ESM.docx]
